# An enriched biosignature of gut microbiota-dependent metabolites characterizes maternal plasma in a mouse model of fetal alcohol spectrum disorder

**DOI:** 10.1038/s41598-020-80093-8

**Published:** 2021-01-08

**Authors:** Manjot S. Virdee, Nipun Saini, Colin D. Kay, Andrew P. Neilson, Sze Ting Cecilia Kwan, Kaylee K. Helfrich, Sandra M. Mooney, Susan M. Smith

**Affiliations:** 1 Department of Nutrition, UNC Nutrition Research Institute, University of North Carolina at Chapel Hill, 500 Laureate Way, Kannapolis, NC 28082 USA; 2 Department of Food Bioprocessing and Nutrition Sciences, Plants for Human Health Institute, North Carolina State University, Kannapolis, NC 28081 USA

**Keywords:** Neurodevelopmental disorders, Disease model, Microbiome, Metabolomics, Functional clustering, Developmental disorders, Metabolomics, Cell death in the nervous system

## Abstract

Prenatal alcohol exposure (PAE) causes permanent cognitive disability. The enteric microbiome generates microbial-dependent products (MDPs) that may contribute to disorders including autism, depression, and anxiety; it is unknown whether similar alterations occur in PAE. Using a mouse PAE model, we performed untargeted metabolome analyses upon the maternal–fetal dyad at gestational day 17.5. Hierarchical clustering by principal component analysis and Pearson’s correlation of maternal plasma (813 metabolites) both identified MDPs as significant predictors for PAE. The majority were phenolic acids enriched in PAE. Correlational network analyses revealed that alcohol altered plasma MDP-metabolite relationships, and alcohol-exposed maternal plasma was characterized by a subnetwork dominated by phenolic acids. Twenty-nine MDPs were detected in fetal liver and sixteen in fetal brain, where their impact is unknown. Several of these, including 4-ethylphenylsulfate, oxindole, indolepropionate, p-cresol sulfate, catechol sulfate, and salicylate, are implicated in other neurological disorders. We conclude that MDPs constitute a characteristic biosignature that distinguishes PAE. These MDPs are abundant in human plasma, where they influence physiology and disease. Their altered abundance here may reflect alcohol’s known effects on microbiota composition and gut permeability. We propose that the maternal microbiome and its MDPs are a previously unrecognized influence upon the pathologies that typify PAE.

## Introduction

Prenatal alcohol exposure (PAE) causes behavioral, growth, and physical anomalies known as Fetal Alcohol Spectrum Disorder (FASD); the deficits in cognition, learning, memory, and executive function persist across the lifespan^1–3^. FASD is a significant public health problem. In the US, an estimated 4.1% (range 3.1–5.0%) of U.S. first graders meet the criteria for an FASD diagnosis^4^, and 3.9% of pregnant women admit to binge drinking in the past 30 days (four or more drinks per occasion)^5^. Unbiased sampling of newborn bloodspots reports even higher exposure rates, and 8.4% in a Texas-statewide sample tested positive for PAE during the month prior to birth^6^. Despite these high rates of prevalence, implementation of screening to identify alcohol-exposed pregnancies remains challenging for complex reasons. Social stigmas surrounding gestational alcohol consumption discourage accurate self-disclosure^7^. Biomarkers such as ethyl-glucuronide and phosphatidylethanol have diagnostic utility^8,9^, but their interpretation is complicated by modifying factors that include the level, pattern, and timing of drinking, genetics, nutritional status, and maternal body mass index^10–13^. A clearer understanding of alcohol-related biomarkers would inform their development and interpretation, and could be leveraged into interventions that attenuate alcohol’s damage.

One candidate modifier of FASD that receives little attention is the microbiome. The enteric microbiome modulates host activity, in part, through its generation of small molecules; these microbial-dependent products (MDPs) include direct products of microbial metabolism (volatile fatty acids, indoles), microbial action upon host-derived metabolites (secondary bile acids), and compounds liberated from the food matrix by microbial digestion (phytochemicals). These are absorbed predominantly through the colon and circulate at physiologically relevant concentrations (nanomolar to high micromolar), where they act as high-affinity ligands for signaling systems that govern diverse processes including bile acid synthesis, gut function, insulin sensitivity, immune function, and vascular health^14–18^. Relevant for FASD, dysfunction of the enteric microbiome has been implicated in neurological disorders including Alzheimer’s disease, amyotrophic lateral sclerosis, autism spectrum disorder, depression, multiple sclerosis, Parkinson’s disease, and seizure risk^14,19–24^. Various MDPs have been shown to interact with the nervous system at physiologically relevant concentrations. For example, microbial-derived indoles cross the blood–brain barrier to modulate motor, anxiety, and other behaviors, as well as microglial-mediated neuroinflammation^25–28^. The microbial-derived polyphenol 3,4-dihydroxyphenylacetate modulates dopamine and catecholamine metabolism and clearance^24,28,29^. Mechanistically, fecal transfers from diseased mice can recreate the MDP signatures, pathologies, and select characteristics of neurological disorders including autism, depression, Parkinson’s disease, amyotrophic lateral sclerosis, and ketogenic refractory epilepsy^19,21,23,30–32^.

Although microbial dysfunction is causative in alcohol-related diseases such as cirrhosis and pancreatitis, potential contributions to FASD are unknown. Alcohol reduces the immunological activities and mucosal tight junctions that maintain the intestinal barrier’s integrity, and thus enhances paracellular entry of MDPs into the circulation^33,34^. Alcohol also alters the enteric microbiome composition and promotes the growth of gram-negative facultative anaerobes that produce exotoxins such as lipopolysaccharide^35–38^. These signal through the toll-like receptors (TLR) to stimulate the inflammation, fibrosis, and cell death that underlie alcoholic end-organ damage. Microbiome contributions to FASD were indirectly suggested by recent demonstrations that loss-of-function in TLR4, which mediates inflammatory responses to microbial LPS, attenuates neuroinflammatory responses to improve memory, anxiety and social behaviors in mouse models of PAE^39,40^. However, it is unknown whether PAE alters the maternal enteric microbiome and the spectrum of biochemicals that it generates, whether these reach the fetus, and how such changes contribute to the pathologies of FASD.

To gain insight into this question, we employed an untargeted UPLC–MS/MS approach and characterized the MDP profile of mother and fetus, using our established mouse model of PAE^41^. We report here that MDPs comprise a distinctive and significant biosignature that distinguishes alcohol-exposed dams from their controls. We readily detect MDPs within the fetus, where PAE again alters their abundance. Several of these analytes were previously implicated in neurological dysfunction. Data implicate MDPs as a hitherto unappreciated contributor to FASD.

## Results

### Litter characteristics

The alcohol dose used (3 g/kg) caused a mean blood alcohol concentration of 211 ± 14 mg/dl at 30 min post-gavage, and the mice were inebriated but did not pass out. Alcohol exposure (ALC) did not affect maternal food intake^41^ or overall weight gain (Supplementary Table S1 online); the ALC dams had a non-significant trend to reduced weight gain during the alcohol exposure period (embryonic day (E) 8.5–E17.5) compared to controls (CON, 11.22 ± 0.47 g; ALC, 9.98 ± 0.44 g; *p* < 0.07). Prenatal alcohol exposure did not affect litter size (*p* = 0.31) or fetal survival (*p* = 0.69) at E17.5, and no adverse outcomes were observed in dam or fetus.

### Alcohol-exposed maternal plasma is enriched in MDPs

**Figure 1:**
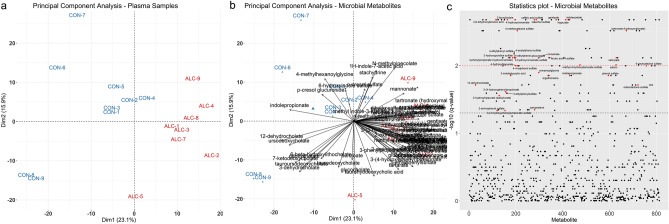
Plasma microbial-derived metabolites distinguish alcohol-exposed and control dams. (**a**) Dimension 1 and 2 of the PCA on the scaled metabolite profiles within plasma of nine control (CON) and eight alcohol-exposed (ALC) dams. (**b**) PCA biplot with microbial-derived metabolites overlaid onto the samples plot. PCA values for the MDPs are presented in Supplemental Table S2. (**c**) T-statistic plot of all 813 metabolites (arranged alphabetically along the x-axis) against their log10 *q*-values. The black dashed line indicates the cut-off for the FDR adjusted value of *q* < 0.05, and the red dashed line indicates *q* < 0.01. Red dots indicate microbial-derived metabolites having *q* < 0.05. Sample size is n = 9 control and n = 8 alcohol-exposed dams.

Untargeted metabolite analysis identified 813 biochemicals in maternal plasma, 733 with known chemical structures and 80 that were unknown. Of the 813 metabolites, 146 had significantly altered representation (q < 0.05 by Mann–Whitney U-test followed by Benjamini–Hochberg correction) in response to PAE. Principle Component Analysis (PCA) of the metabolite profiles showed that alcohol-exposure is a clear driver of variance within the metabolic profiles, and placed one dam (ALC-6) as an outlier (Supplementary Figure S1A,B online). This dam’s plasma ethyl glucuronide level was just 14.0% of that for the other alcohol-exposed dams, suggesting a gavage error, and she was removed from further analysis. Repeating the PCA with omission of ALC-6 revealed that the metabolomic profile explained the separation of the samples by intervention. PC1 explained 23.1% of the sample variance, and PC2 explained an additional 15.9% (Fig. 1a), and visual inspection of the data set revealed that MDPs were among the strongest drivers of PC1 and PC2 (Fig. 1b). Analysis of the log-fold change *q*-values using T-statistic similarly found that MDPs were over-represented (Fig. 1c), and of 146 metabolites having a *q* ≤ 0.05, 28.1% (N = 41) were MDPs, although they comprised 10.5% of the 733 known metabolites. Housing assignment can also affect enteric microbiome composition^42^; remapping the PCA results against housing assignment affirmed that cage assignment did not influence analyte distribution or abundance (Supplemental Fig. S1C).

Of the 70 MDPs detected within control and ALC maternal plasma, 41 (58.6%) had significantly altered representation (*q* ≤ 0.05) in response to alcohol (Table 1); the preponderance (44) were enriched by alcohol-exposure and only two were significantly reduced. The majority of the MDPs (36/70) were plant phenolics that originated from the microbial-mediated fermentation of ingested lignins in the cage bedding^18^, and potentially from starch- or cellulose-bound flavonoids in the purified diet^43^. Alcohol exposure significantly enriched the abundance of 29 plant-derived phenolics, including the alcoholic β-glucoside salicin (12.98-fold), catechol sulfate (7.41-fold), cinnamate (5.28-fold), ferulic acid 4-sulfate (4.50-fold), hippurate (3.37-fold), caffeic acid sulfate (3.21-fold), phenyl sulfate (3.20-fold), and salicylate (3.11-fold). None had reduced abundance, and the overall pattern was one in which ALC significantly increased the plasma abundance of plant-derived aromatics and their phase-II metabolites.

**Table 1:** Microbial-derived products detected in maternal plasma.

Metabolite	HMDB ID	F–C	*p*-Value	*q*-Value	Rel. abundance		%Pos samples	
		Alc/Cont			Cont	Alc	Cont	Alc
**Plant Phenolics**								
Benzoate	HMDB01870	1.07	0.2359	0.4631	958,546 ± 215,335	1,078,999 ± 62,041	100	100
Salicylate	HMDB01895	3.11	0.0006	0.0076	2.14 ± 0.57E+07	7.70 ± 3.01	100	100
Gentisate	HMDB00152	3.99	0.0001	0.0021	225,041 ± 189,114	1,225,172 ± 518,151	100	100
2,6-Dihydroxybenzoic acid	HMDB13676	2.54	0.0001	0.0021	5.42 ± 0.79E+07	1.22 ± 2.95E+07	100	100
Methyl-4-hydroxybenzoate sulfate		3.13	0.0006	0.0097	20,506 ± 0	73,411 ± 40,088	11	88
2-Aminophenol sulfate	HMDB61116	1.89	0.0082	0.0462	29,136 ± 2730	70,453 ± 13,683	44	88
Hippurate	HMDB00714	3.37	0.0001	0.0021	3.16 ± 2.05E+07	11.6 ± 5.27E+07	100	100
2-Hydroxyhippurate	HMDB00840	2.91	0.0040	0.0277	127,409 ± 15,397	295,330 ± 251,375	56	100
4-Hydroxyhippurate	HMDB13678	2.68	0.0010	0.0100	143,377 ± 82,882	387,206 ± 51,704	100	100
Catechol sulfate	HMDB59724	7.41	0.0001	0.0021	491,694 ± 234,179	4,874,352 ± 2,650,700	100	100
4-Methylcatechol sulfate		3.11	0.0002	0.0030	1.20 ± 0.40E+07	2.94 ± 1.09E+07	100	100
4-Vinylcatechol sulfate		3.91	0.0001	0.0021	252,230 ± 207,416	645,391 ± 188,414	100	100
4-Methylbenzenesulfonate		1.05	0.6058	0.8168	117,042 ± 7652	133,763 ± 17,266	100	100
p-Cresol sulfate	HMDB11635	1.03	1.0000	1.0000	1.32 ± 0.29E+08	1.29 ± 0.19 + 08	100	100
p-Cresol glucuronide	HMDB11686	0.80	0.1389	0.3169	6.17 ± 1.32E+07	4.40 ± 1.85E+07	100	100
Phenol sulfate	HMDB60015	3.20	0.0016	0.0139	4.25 ± 3.36E+07	15.45 ± 7.43E+07	100	100
4-Acetylphenol sulfate		7.69	0.0004	0.0066	53,565 ± 12,600	254,938 ± 162,332	22	100
3-Ethylphenylsulfate	HMDB62721	2.05	0.0006	0.0076	144,522 ± 52,789	265,503 ± 84,944	100	100
4-Ethylphenylsulfate	HMDB62551	2.11	0.0037	0.0255	800,922 ± 448,872	1,720,531 ± 671,132	100	100
4-Ethylphenol glucuronide		3.28	0.0006	0.0076	44,781 ± 22,943	156,697 ± 26,135	78	100
4-Vinylphenol sulfate	HMDB62775	1.91	0.0010	0.0100	4.76 ± 2.20E+07	9.46 ± 0.26E+07	100	100
3-Phenylpropionate	HMDB00764	3.69	0.0006	0.0076	0.56 ± 0.32E+07	1.96 ± 1.23E+07	100	100
3-(3-Hydroxyphenyl)propionate sulfate		2.61	0.0083	0.0462	121,465 ± 0	220,591 ± 197,731	11	88
3-(4-Hydroxyphenyl)propionate	HMDB02199	3.45	0.0031	0.0244	88,159 ± 49,748	266,463 ± 196,354	67	100
Cinnamate	HMDB00930	5.28	0.0006	0.0076	57,488 ± 14,984	292,667 ± 156,654	89	100
Cinnamoylglycine	HMDB11621	4.86	0.0013	0.0126	190,747 ± 48,511	767,472 ± 534,399	89	100
4-Hydroxycinnamate	HMDB02035	7.23	0.0001	0.0021	0.37 ± 0.26E+07	1.94 ± 0.40E+07	100	100
4-Hydroxycinnamate sulfate		7.57	0.0006	0.0076	118,831 ± 57,335	435,877 ± 217,416	78	100
Caffeic acid sulfate	HMDB41708	3.21	0.0001	0.0021	6.86 ± 5.08E+07	2.08 ± 5.66E+07	100	100
Dihydrocaffeate sulfate		1.44	0.0592	0.1859	124,031 ± 45,309	17,689 9 ± 35,472	100	100
4-Allylphenol sulfate		2.53	0.0139	0.0680	283,211 ± 367,729	562,278 ± 71,665	78	100
Ferulic acid 4-sulfate	HMDB29200	4.50	0.0005	0.0076	64,588 ± 33,678	270,978 ± 56,994	56	100
Salicin	HMDB03546	12.98	0.0006	0.0097	83,702 ± 86,337	363,162 ± 363,699	33	100
Enterolactone		1.64	0.0016	0.0139	0.89 ± 3.93E+07	1.44 ± 0.24E+07	100	100
Enterolactone sulfate		5.32	0.0025	0.0183	43,661 ± 19,659	313,537 ± 289,225	100	100
Thymol sulfate	HMDB01878	1.96	0.2739	0.4964	48,567 ± 6615	41,271 ± 20,617	33	75
**Indole derivatives**								
1H-Indole-7-acetic acid		1.32	0.5634	0.7981	79,213 ± 20,397	116,675 ± 89,689	89	100
2-Oxindole-3-acetate	HMDB35514	1.32	0.1315	0.3170	43,730 ± 12,970	46,591 ± 11,210	22	75
3-Formylindole	HMDB29737	1.50	0.0001	0.0021	6.70 ± 1.01E+07	9.55 ± 0.65E+07	100	100
3-Indoleglyoxylic acid		1.48	0.0016	0.0139	5.37 ± 0.71E+07	6.74 ± 1.32	100	100
6-Hydroxyindole sulfate		0.99	1.0000	1.0000	324,828 ± 69,170	312,948 ± 117,634	100	100
Indoleacetate	HMDB00197	1.46	0.0001	0.0021	1.12 ± 0.11E+07	1.67 ± 0.25	100	100
Indolelactate	HMDB00671	1.76	0.0001	0.0021	2.91 ± 0.58E+08	4.85 ± 0.96E+08	100	100
Indolepropionate	MMDB02302	0.41	0.0016	0.0139	1.28 ± 0.56E+08	0.67 ± 0.27E+08	100	100
Indolin-2-one		1.47	0.0152	0.0680	307,433 ± 108,591	468,992 ± 104,000	100	100
Methyl indole-3-acetate	HMDB29738	0.77	0.5414	0.7682	731,266 ± 192,684	647,994 ± 338,340	100	100
**Sugars and derivatives**								
Erythritol	HMDB02994	1.10	0.6730	0.8549	386,441 ± 51,606	407,249 ± 115,312	100	100
Gluconate	HMDB00625	1.39	0.0744	0.2131	1.55 ± 0.23E+07	2.08 ± 0.55E+07	100	100
Mannonate		1.21	0.6730	0.8549	6.10 ± 2.15E+07	7.34 ± 1.11E+07	100	100
Ribitol	HMDB00508	2.23	0.0037	0.0255	504,272 ± 55,398	845,683 ± 468,022	100	100
Tartarate	HMDB00956	1.59	0.0274	0.1017	2.30 ± 1.13E+08	3.22 ± 1.02E+08	100	100
Tartronate (hydroxymalonate)	HMDB35227	1.68	0.0001	0.0021	1.52 ± 0.21E+07	2.44 ± 0.23E+07	100	100
Threonate	HMDB00943	1.86	0.0001	0.0021	4.04 ± 1.04E+08	7.19 ± 1.26E+08	100	100
**Betaines**								
Ergothioneine	HMDB03045	1.92	0.0016	0.0139	1.23 ± 0.30E+07	2.35 ± 1.09	100	100
Hercynine		1.99	0.0272	0.1017	340,969 ± 0	227,104 ± 118,864	11	88
Stachydrine	HMDB04827	0.92	0.1139	0.2850	1.46 ± 0.64E+07	2.21 ± 1.00E+07	100	100
**Plant sterols**								
Beta-sitosterol	HMDB00852	1.40	0.0111	0.0557	0.72 ± 0.22E+07	1.01 ± 0.21	100	100
Campesterol	HMDB02869	1.28	0.0079	0.0449	2.00 ± 0.61E+07	2.64 ± 0.26	100	100
**Secondary bile acids**								
3-Dehydrocholate	HMDB00502	0.09	0.5911	0.8168	2.39 ± 3.21E+07	0.08 ± 0.05	33	38
6-Beta-hydroxylithocholate	HMDB00811	0.35	0.7430	0.8818	52,548 ± 37,161	126,467 ± 56,019	100	100
7-Ketodeoxycholate	HMDB00391	0.15	0.2766	0.4964	1.37 ± 1.24E+07	0.94 ± 0.60E+07	100	100
12-Dehydrocholate	HMDB00400	0.02	0.0023	0.0193	152,421 ± 178,862	94,362 ± 0	100	12
Deoxycholate	HMDB00626	0.43	0.4807	0.7171	7.72 ± 0.63E+07	4.71 ± 1.96E+07	100	100
Glycocholate	HMDB00138	1.47	0.1603	0.3577	138,596 ± 160,488	289,158 ± 259,334	67	10
Taurodeoxycholate	HMDB00896	0.48	0.1139	0.2850	1.23 ± 0.91E+07	3.12 ± 1.79E+07	100	100
Tauroursodeoxycholate	HMDB00874	0.26	0.8148	0.9162	2.82 ± 1.31E+07	3.86 ± 0.71E+07	100	100
Taurohyodeoxycholic acid		2.21	0.0220	0.0905	0.34 ± 0.38E+07	1.92 ± 1.34E+07	56	100
Ursocholate		2.19	0.0194	0.0850	100,831 ± 15,843	368,247 ± 132,692	33	88
Ursodeoxycholate	HMDB00946	0.18	0.0604	0.1890	2.29 ± 2.05E+07	1.23 ± 0.87E+07	100	88
**Others**								
N-Methylpipecolate		1.13	0.3704	0.6096	472,305 ± 136,660 539,118 ± 64,132	100	100	
3-Hydroxypyridine sulfate		3.57	0.0006	0.0076	40,012 ± 24,268	86,737 ± 44,427	33	100

N = 9 control and N-8 alcohol-exposed dams; *p*-value by Mann–Whitney U-test; *q*-value by Benjamini–Hochberg FDR correction. Relative Abundance presents median ± median absolute deviation (MAD).

*Alc* Alcohol-treated, *Cont* control, *F-C* fold-change.

Alcohol-exposure also increased the maternal plasma levels of multiple indole derivatives including indoleacetate, indolelactate, indolin-2-one, 3-formylindole, and 3-indoleglyoxylic acid (range 1.47–1.76-fold), and decreased the abundance of indolepropionate (0.41-fold). It also altered the abundance of secondary bile acids, which are generated by microbial action upon those primary bile acids not resorbed in the ileum. Of the eleven secondary bile acids detected in maternal plasma, eight were less abundant in ALC dams, although only 12-dehydrocholate was significantly reduced (0.02-fold, q = 0.0193). In contrast, taurohyodeoxycholic acid (2.21-fold) and ursocholate (2.19-fold) were elevated in alcohol-exposed maternal plasma, but not significantly. Also increased were the sugar derivatives ribitol, tartronate, and threonate, and the betaine ergothioneine. Although not microbial-derived, the phytosterols beta-sitosterol and campesterol were also elevated by alcohol-exposure.

### MDPs comprise a significant biosignature in plasma of ALC dams

**Figure 2:**
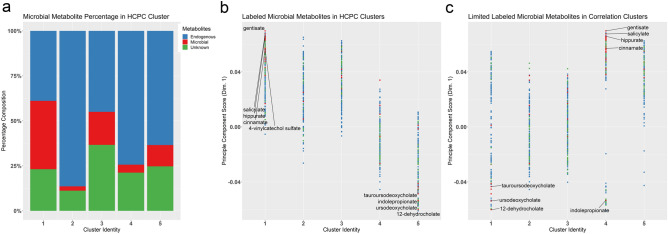
Hierarchical clustering for 813 metabolites in maternal plasma. (**a**) Hierarchical Clustering on Principal Components. Percentage composition of microbial metabolites in the hierarchical clusters of the sample PCA loadings, using Ward’s method. Compounds were defined as microbial-derived (red), endogenous-derived (blue), and unknown identity (green). (**b**) Hierarchical clustering of the sample PCA loadings, plotted against the principle component score for Dimension 1. Plant phenolics were correlated in Cluster 1, and secondary bile acids in Cluster 5; Cluster 3 represented metabolites largely unaffected by alcohol. (**c**) Hierarchical Clustering on Spearman’s Correlation. The microbial-derived metabolites retained their relationships and plant-derived phenolics were correlated in cluster 4, while the secondary bile acids were in cluster 1. The correlation within Clusters 4 and 5 indicate metabolites having an immediate cellular process affected by the alcohol treatment, whereas the former clusters contain metabolites affected more distantly. For (**b**) and (**c**), a positive principal component score indicates the metabolite has increased abundance in response to alcohol; negative scores signify reduced abundance. Sample size is n = 9 control and n = 8 alcohol-exposed dams.

We utilized Hierarchical Clustering of Principal Components (HCPC) to understand the relationships among these metabolite features. This placed the 813 metabolites into five, evenly distributed clusters using Ward’s method. The MDPs were unevenly distributed across the clusters, and a majority segregated into cluster 1 (57.8%, 41 of 71 MDPs), followed by clusters 3 (11/71) and 5 (11/71) (Fig. 2a). Clusters 2 and 4 were dominated by endogenous metabolites. This clustering of MDPs primarily reflected their contribution to PC1, which captured the greatest class separation between ALC and control (Fig. 2b). The MDPs in Cluster 1 were predominantly plant-derived aromatics, and all were enriched in ALC (Table 2; Supplementary Table S2 online). In contrast, the MDPs in cluster 5 were enriched in control plasma and they were mostly (9/11) secondary bile acids. Clusters 3 and 4 were highly skewed towards other Principal Component Dimensions, and repeating the PCA and PLSDA analysis according to PC2 (Supplementary Figure S2 online) suggested that dimension 2 modeled the time of plasma collection following the intervention. This cluster separation was relevant only for the ALC dataset and not the controls. Because this influence did not involve the MDPs, it is the focus of a separate investigation described elsewhere. In summary, the HCPC and PLSDA analysis revealed that the MDPs clustered by treatment response based on the PC loadings. These MDPs had a disproportionate influence in explaining exposure variance within the metabolite dataset, and their influence was defined by their molecular structure, in addition to their relative abundance and p-value.

**Table 2:** MDPs that drive dimensions 1 and 2 in hierarchical clustering.

**Cluster 1 metabolites (N = 41)**
2,6-Dihydroxybenzoic acid, 2-hydroxyhippurate (salicylurate), 3-(3-hydroxyphenyl)propionate sulfate, 3-(4-hydroxyphenyl)propionate, 3-ethylphenylsulfate, 3-formylindole, 3-hydroxypyridine sulfate, 3-indoleglyoxylic acid, 3-phenylpropionate (hydrocinnamate), 4-acetylphenyl sulfate, 4-allylphenol sulfate, 4-ethylphenol glucuronide, 4-ethylphenyl sulfate, 4-hydroxycinnamate, 4-hydroxycinnamate sulfate, 4-methylcatechol sulfate, 4-vinylcatechol sulfate, 4-vinylphenol sulfate, beta-sitosterol, caffeic acid sulfate, campesterol, catechol sulfate, cinnamate, enterolactone, enterolactone sulfate, ergothioneine, ferulic acid 4-sulfate, gentisate, gluconate, hippurate, histidine betaine (hercynine), indoleacetate, methyl-4-hydroxybenzoate sulfate, phenol sulfate, salicin, salicylate, tartarate, tartronate (hydroxymalonate), taurohyodeoxycholic acid, threonate, ursocholate
**Cluster 2 metabolites (N = 3)**
1H-indole-7-acetic acid, 4-methylbenzenesulfonate, methyl indole-3-acetate
**Cluster 3 metabolites (N = 11)**
2-Aminophenol sulfate, 2-oxindole-3-acetate, 4-hydroxyhippurate, cinnamoylglycine, dihydrocaffeate sulfate, erythritol, indolelactate, indolin-2-one, mannonate, ribitol, thymol sulfate
**Cluster 4 metabolites (N = 5)**
6-Hydroxyindole sulfate, N-methylpipecolate, p-cresol glucuronide, p-cresol sulfate, stachydrine
**Cluster 5 metabolites (N = 11)**
12-Dehydrocholate, 3-dehydrocholate, 6-beta-hydroxylithocholate, 7-ketodeoxycholate, benzoate, deoxycholate, glycocholate, indolepropionate, taurodeoxycholate, tauroursodeoxycholate, ursodeoxycholate

The PCA loadings for these metabolites are presented in Supplemental Table S2, and are depicted in Fig. 2b. N = 9 control and N = 8 alcohol-treated dams.

Correlation analysis further informs whether influences in addition to exposure drive the metabolite relationships and variance across the dataset. We repeated the hierarchical clustering using Spearman’s correlation (Fig. 2c). The strong association between the plant phenolics was retained, and 47/71 were correlated within Cluster 5, comprising 27.8% of that cluster, and these were all enriched by alcohol exposure (Table 3, Supplementary Table S3). New associations also emerged, and Cluster 1 (13/71) included a mixture of phenolics, indoles, and secondary bile acids largely unaffected by alcohol-exposure. These findings further support that alcohol exposure strongly influenced plasma MDP content, and their relationships further depended on chemical structure and metabolic fate.

**Table 3:** MDPs by cluster from the hierarchical clustering on Spearman’s correlation.

**Cluster 1 metabolites (N = 13)**
1H-indole-7-acetic acid, 4-methylbenzenesulfonate, 6-beta-hydroxylithocholate, 6-hydroxyindole sulfate, benzoate, glycocholate, mannonate, N-methylpipecolate, stachydrine, taurodeoxycholate, taurohyodeoxycholic acid, tauroursodeoxycholate, thymol sulfate
**Cluster 2 metabolites (N = 3)**
Methyl indole-3-acetate, p-cresol glucuronide, p-cresol sulfate
**Cluster 3 metabolites (N = 5)**
3-Dehydrocholate, 7-ketodeoxycholate, deoxycholate, histidine betaine (hercynine), ursodeoxycholate
**Cluster 4 metabolites (N = 5)**
2-Aminophenol sulfate, 2-oxindole-3-acetate, dihydrocaffeate sulfate, erythritol, indolin-2-one
**Cluster 5 metabolites (N = 45)**
12-Dehydrocholate, 2,6-dihydroxybenzoic acid, 2-hydroxyhippurate (salicylurate), 3-(3-hydroxyphenyl)propionate sulfate, 3-(4-hydroxyphenyl)propionate, 3-ethylphenylsulfate, 3-formylindole, 3-hydroxypyridine sulfate, 3-indoleglyoxylic acid, 3-phenylpropionate (hydrocinnamate), 4-acetylphenyl sulfate, 4-allylphenol sulfate, 4-ethylphenol glucuronide, 4-ethylphenyl sulfate, 4-hydroxycinnamate, 4-hydroxycinnamate sulfate, 4-hydroxyhippurate, 4-methylcatechol sulfate, 4-vinylcatechol sulfate, 4-vinylphenol sulfate, beta-sitosterol, caffeic acid sulfate, campesterol, catechol sulfate, cinnamate, cinnamoylglycine, enterolactone, enterolactone sulfate, ergothioneine, ferulic acid 4-sulfate, gentisate, gluconate, hippurate, indoleacetate, indolelactate, indolepropionate, methyl-4-hydroxybenzoate sulfate, phenol sulfate, ribitol, salicin, salicylate, tartarate, tartronate (hydroxymalonate), threonate, ursocholate

Spearman’s coefficients for each metabolite are presented in Supplemental Table S3, and are depicted in Fig. 2c. N = 9 control and N = 8 alcohol-treated dams.

**Figure 3:**
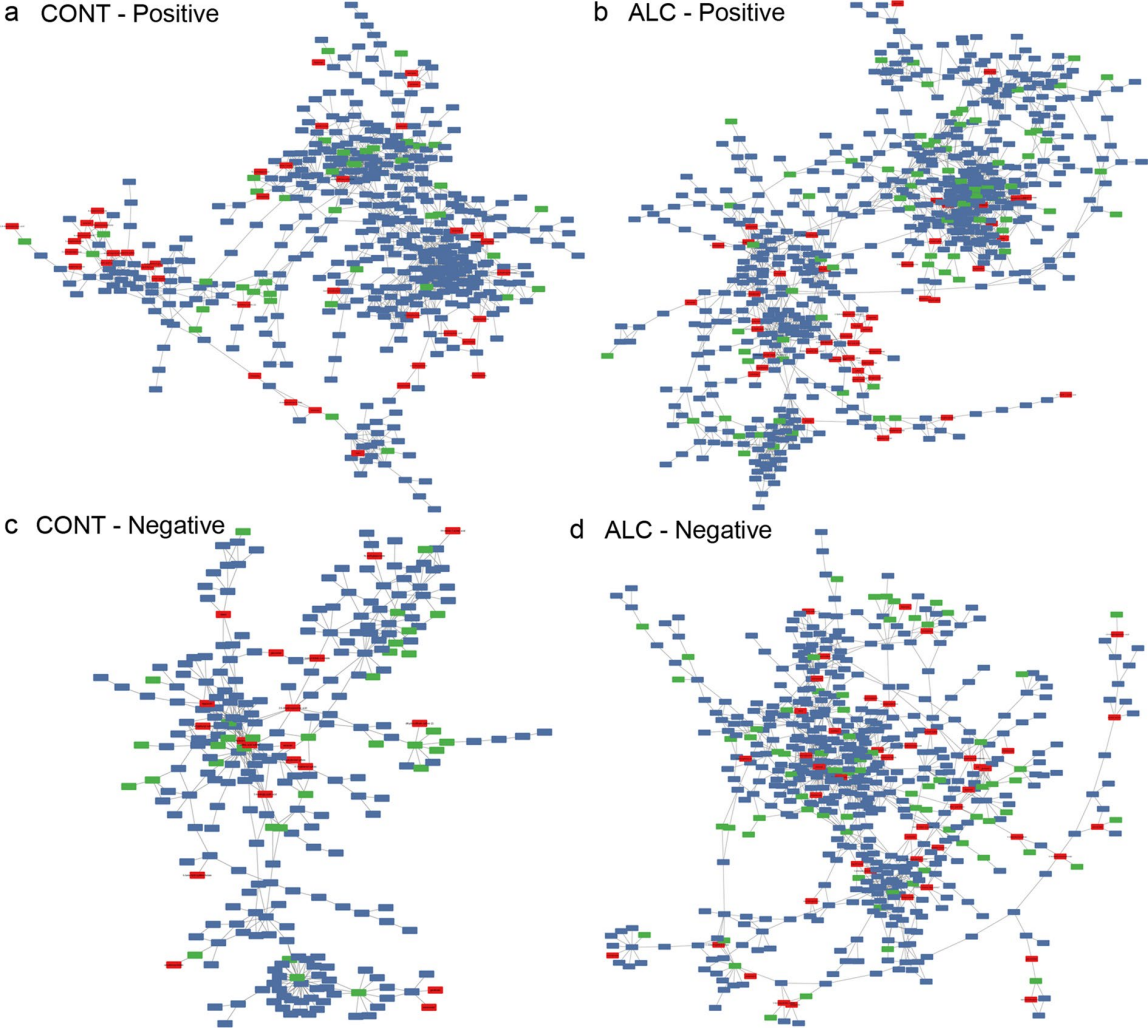
Correlation analysis identifies microbial metabolite networks that are at statistical equilibrium within maternal plasma. Images were generated in Cytoscape (version 3.7.2) using R-Cy3 (version 2.6.3), wherein the network edges represent between-node Spearman’s correlations > 0.90 (**a**,**b**) or ≥ − 0.90 (**c**,**d**); distance between nodes indicates strength of interaction. Colors as in Fig. 2; red indicates MDPs, blue indicates endogenous compounds, and green are unknown compounds. (**a**) Positive correlation network of metabolites in control (CONT) maternal plasma having Spearman’s values ≥ 0.90. (**b**) Positive correlation network of metabolites in alcohol-exposed (ALC) maternal plasma having Spearman’s values ≥ 0.90. (**c**) Negative correlation network of metabolites in control (CONT) maternal plasma having Spearman’s values ≥ − 0.90. (**d**) Negative correlation network of metabolites in alcohol-exposed (ALC) maternal plasma having Spearman’s values ≥ − 0.90. Sample size is n = 9 control and n = 8 alcohol-exposed dams.

Metabolite classes that tightly cluster together in the correlation analysis share a consistent response to treatment. Similarly, metabolites that are downstream products of a shared cellular process affected by alcohol also will maintain equilibrium with each other during the analysis. Thus, highly conserved correlations likely represent a metabolite set that exists within a molecular equilibrium. The consistent clustering of MDPs in both the HCPC and Spearman’s correlation suggested the existence of such relationships. To investigate this, we filtered the correlation matrix to correlations greater than 0.9, and subjected these to network correlation analysis, and segregated by treatment. This yielded very different network structures for Control and ALC maternal plasma (Fig. 3). The network architecture of the controls was comprised of two dense hubs connected by tightly-linked interactions, and each joined to separate satellites that were in turn weakly linked (Fig. 3a). In contrast, the ALC plasma network architecture was dominated by just a single dense hub that was more loosely connected with a second, more diffuse hub, each with an adjoining smaller satellite (Fig. 3b). The MDPs held quite different relationships within these two architectural structures, and the plant phenols formed a dense mini-structure in ALC, whereas no such network appeared in controls; the latter’s largest MDP set was a mix of phenolics, indoles, and sugar acids. A parallel analysis that focused on Spearman correlations less than 0.9 revealed similarly divergent networks, such that the Control network (Fig. 3c) featured far fewer metabolites than did ALC (Fig. 3d), signaling that the plasma metabolite profile of ALC was characterized by a loss of tight regulatory control. This is endorsed by the more dispersed structure of the ALC network and suggests an overall weakening of metabolite relationships that may be a product of dysregulated metabolism. Overall, the analyses revealed that alcohol-exposure altered the relationships between MDPs and endogenous metabolites, and endorsed the MDP biosignature for alcohol-exposed maternal plasma.

**Figure 4:**
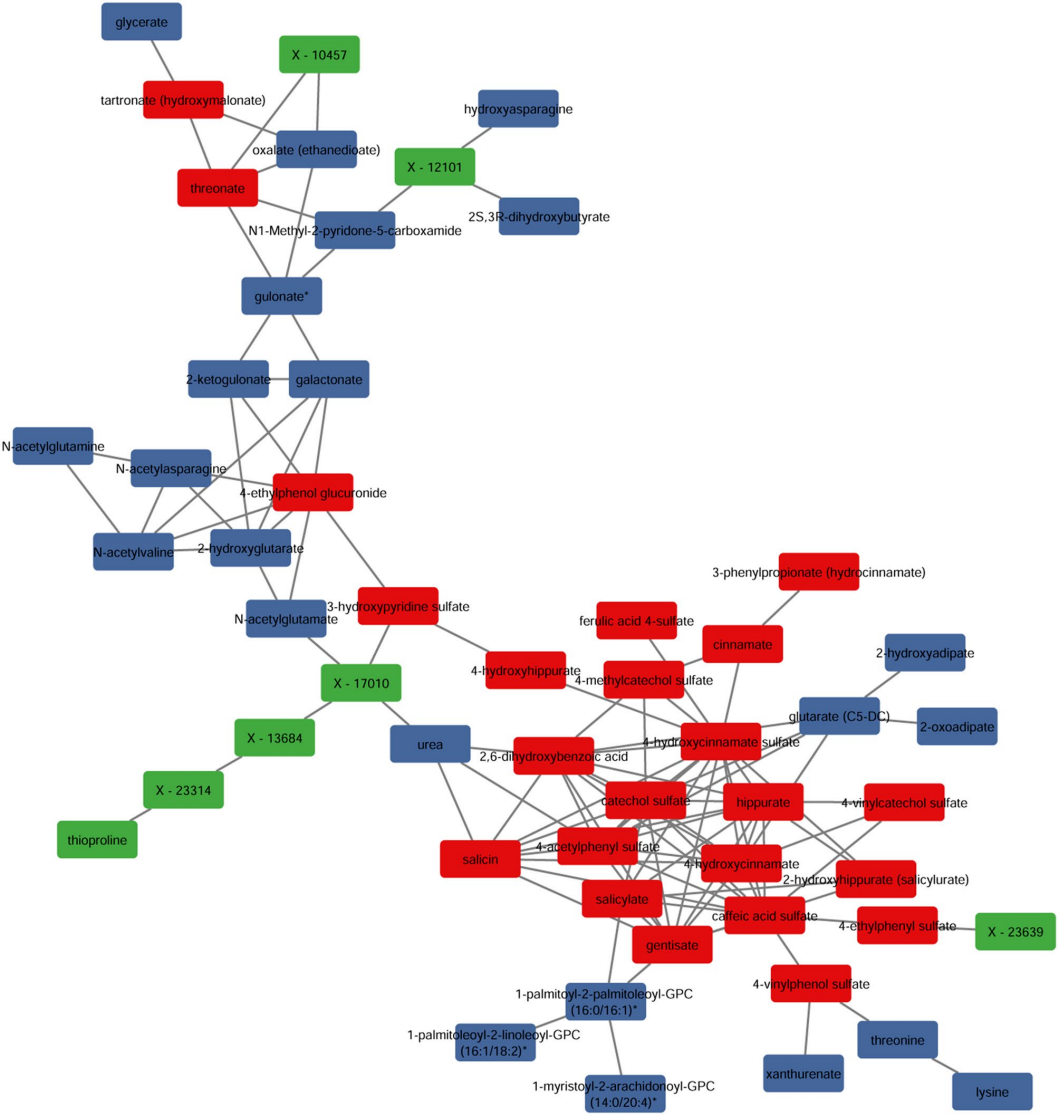
Positive correlation analysis identifies a network that is enriched in microbial metabolites and is at statistical equilibrium within maternal plasma. Images were generated in Cytoscape (version 3.7.2) using R-Cy3 (version 2.6.3), wherein the network edges represent between-node Spearman’s correlations > 0.90; the distance between nodes indicates strength of interaction. Colors as in Fig. 2; red indicates MDPs, blue indicates endogenous compounds, and green are unknown compounds. The compound X-17010 is likely the MDP 4-vinylcatechol sulfate, based on its molecular mass.

Additional insight was obtained by merging the Control and Alcohol plasma datasets, and again performing network analysis filtered by the Spearman’s correlations. For the negative correlations, this only yielded two-node subnetworks, none of which contained MDPs. For the positively correlated metabolites, this yielded a network dominated by MDPs enriched in ALC (Fig. 4). This included a tightly correlated subnetwork of nineteen plant phenolics (hippurates, catechols, salicins, phenols) and a smaller, linked subnetwork of sugar acids that was further linked with endogenous-derived sugar acids. Also included were several unknowns including one tentatively identified as the plant-derived phenolic pyrocatechol sulfate (X-17010), based on its fragmentation mass. The tight relationship between these phenolic MDPs suggested they were at equilibrium with each other in response to alcohol, and further suggested that that their enrichment shared a similar or conserved molecular process or cause.

### MDPs are enriched in the PAE fetus

**Figure 5:**
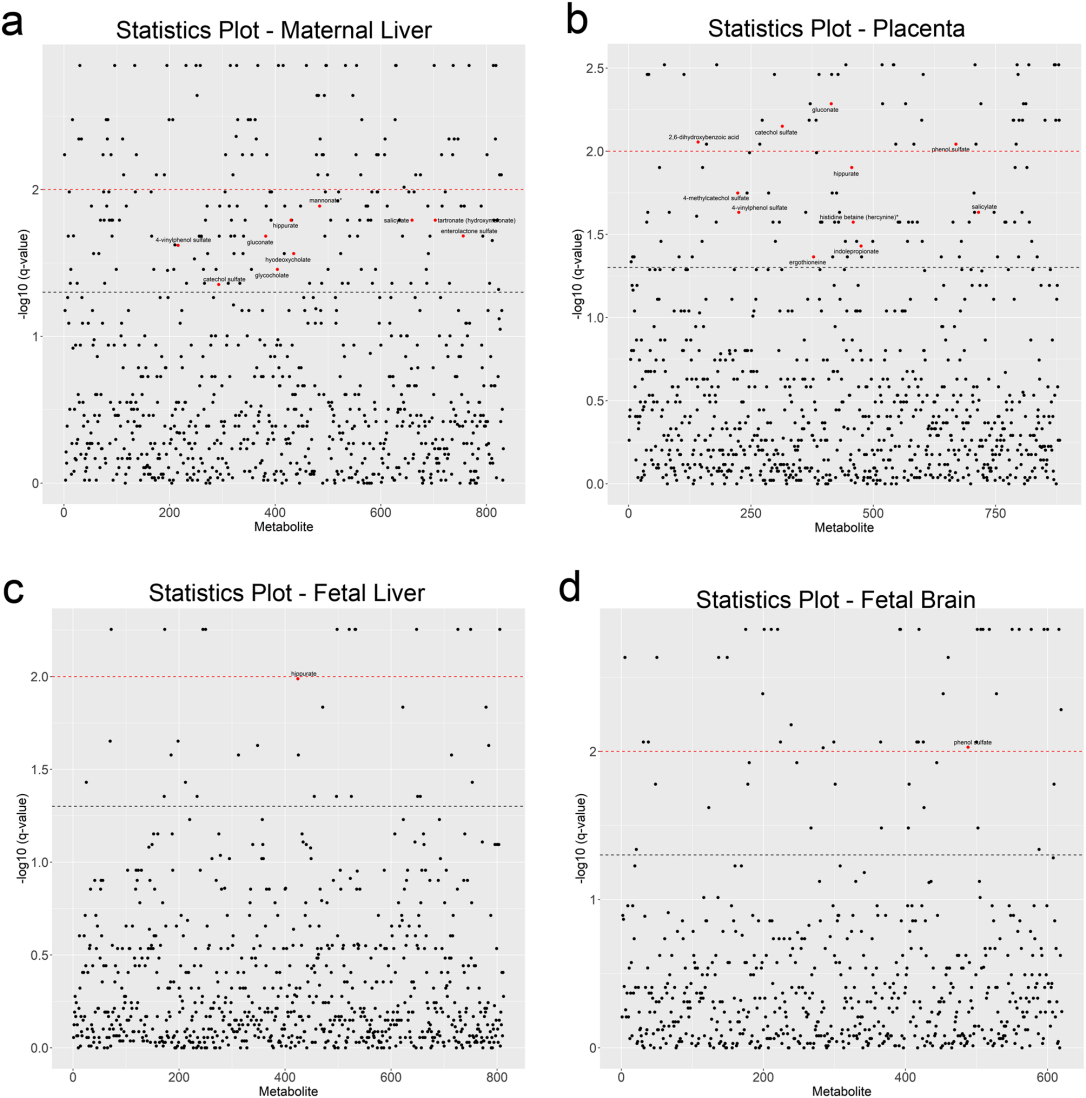
T-Statistic plot of all metabolites present in maternal and fetal tissues, plotted against their − log10 *q*-values. Metabolites are arranged along x-axis in alphabetical order. The black dashed line indicates the cut-off for the FDR adjusted value of *q* < 0.05, and the red dashed line indicates *q* < 0.01. Red dots indicate microbial-derived metabolites having *q* < 0.05. (**a**) T-statistic plot of the 854 metabolites detected in maternal liver. (**b**) T-statistic plot of the 881 metabolites detected in placenta. (**c**) T-statistic plot of the 854 metabolites detected in fetal liver. (**d**) T-statistic plot of the 621 metabolites detected in fetal brain. Sample size is n = 9 control and n = 8 alcohol-exposed dams.

We asked if this ALC-dependent MDP biosignature extended to other maternal–fetal compartments. Five additional MDPs were detected in these tissues (kojibiose, beta-guanidinopropanoate, hyodeoxycholate, taurohyocholate, and taurolithocholate), for a total of 75 MDPs. Many of the plasma MDPs were phase-II conjugates, synthesized primarily by enterocytes and hepatocytes to facilitate their urinary (sulfate and glucuronide) or biliary (O-methyl) excretion. However, the MDP profile of maternal liver (32/75) significantly differed from that of plasma (Table 4) and was dominated by secondary bile acids and sugar derivatives; far fewer plant phenolics were detected (10 vs. 36). Fold-changes in response to alcohol were modest, and just 10 of 32 (31.2%) hepatic MDPs had significantly altered representation in ALC. Analysis in the T-statistic (Fig. 5a) affirmed their lower representation among the significantly altered biofeatures in maternal liver (10/205), suggesting that MDPs were not major drivers of metabolite variance in response to alcohol for this tissue.

**Table 4:** Comparison of MDP profiles and their abundance in maternal plasma and liver, placenta, and fetal liver and brain.

Biochemical name	Maternal plasma		Maternal liver		Placenta		Fetal liver		Fetal brain	
	F–C	q-value	F–C	*q*-value	F–C	*q*-value	F–C	*q*-value	F–C	*q*-value
Benzoate	1.07	0.4631	0.51	0.4090	–		0.92	1.0000	1.21	0.4628
Salicylate	3.11	0.0076	2.60	0.0161	2.03	0.0233	1.46	0.2917	–	
Gentisate	3.99	0.0021	–		–		–		–	
2,6-Dihydroxybenzoic acid	2.54	0.0021	–		2.04	0.0088	–		–	
Methyl-4-hydroxybenzoate-SO_4_	3.13	0.0097	–		–		–		–	
2-Aminophenol sulfate	1.89	0.0082	–		–		–		–	
Hippurate	3.37	0.0001	1.97	0.0161	2.31	0.0125	2.43	0.0103	1.82	0.0656
2-Hydroxyhippurate	2.91	0.0040	–		–		–		–	
4-Hydroxyhippurate	2.68	0.0010	–		–		–		–	
catechol sulfate	7.41	0.0001	2.60	0.0443	6.62	0.0071	1.83	0.1381	–	
4-Methylcatechol sulfate	3.11	0.0002	–		3.11	0.0178	–		–	
4-Vinylcatechol sulfate	3.91	0.0021	1.25	0.4090	–		1.02	0.9347	0.83	0.3213
4-Methylbenzenesulfonate	1.05	0.8168	–		–		–		–	
p-Cresol sulfate	1.03	1.0000	1.11	0.6363	0.93	0.6270	0.78	0.2917	0.81	0.2378
p-Cresol glucuronide	0.80	0.3169	0.77	0.3139	0.74	0.2932	0.86	0.6422	–	
Phenol sulfate	3.20	0.0139	1.42	0.0813	–		1.34	0.0704	2.72	0.0094
4-Acetylphenol sulfate	7.69	0.0066	–		–		–		–	
3-Ethylphenylsulfate	2.05	0.0076	–		–		–		–	
4-Ethylphenylsulfate	2.11	0.0255	–		1.52	0.0515	–		–	
4-Ethylphenol glucuronide	3.28	0.0076	–		–		–		–	
4-Vinylphenol sulfate	1.91	0.0010	2.06	0.0240	1.39	0.0233	1.03	0.8185	–	
3-Phenylpropionate	3.69	0.0076	–		–		–		–	
3-(3Hydroxyphenyl)propionate-SO_4_	2.61	0.0462	–		–		–		–	
3-(4-Hydroxyphenyl)propionate	3.45	0.0244	–		–		–		–	
Cinnamate	5.28	0.0076	–		–		–		–	
Cinnamoylglycine	4.86	0.0126	–		–		–		–	
4-Hydroxycinnamate	7.23	0.0021	–		–		–		–	
4-Hydroxycinnamate sulfate	7.57	0.0076	–		–		–		–	
Caffeic acid sulfate	3.21	0.0021	–		–		–		–	
Dihydrocaffeate sulfate	1.44	0.1859	–		–		–		–	
4-Allylphenol sulfate	2.53	0.0680	–		–		–		–	
Ferulic acid 4-sulfate	4.50	0.0076	–		–		–		–	
Salicin	12.98	0.0097	–		–		–		–	
Enterolactone	1.64	0.0139	–		–		–		–	
Enterolactone sulfate	5.32	0.0183	4.76	0.0207	–		–		–	
Thymol sulfate	1.96	0.4964	–		–		–		–	
Indoleacetate	1.46	0.0021	0.94	0.7303	0.94	0.6270	0.94	0.8185	1.26	0.3213
Indolelactate	1.76	0.0021	–		1.15	0.1580	1.64	0.01653	0.99	0.3911
Indolepropionate	0.41	0.0139	–		0.29	0.0371	–		–	
3-Formylindole	1.50	0.0021	–		1.18	0.1802	–		0.94	0.9689
Methyl indole-3-acetate	0.77	0.7682	–		–		–		–	
3-Indoleglyoxylic acid	1.48	0.0139	–		1.17	0.8252	–		–	
Indolin-2-one	1.47	0.0680	–		–		–		–	
2-Oxindole-3-acetate	1.32	0.3170	–		–		–		–	
1H-indole-7-acetic acid	1.32	0.7981	–		–		–		–	
6-Hydroxyindole sulfate	0.99	1.0000	–		–		–		–	
Kojibiose	–		0.82	0.4090	–		1.61	0.3733	–	
Mannonate	1.21	0.8549	0.68	0.0129	1.13	0.3219	1.04	0.9183	1.06	0.5390
Gluconate	1.39	0.2131	0.65	0.0207	0.50	0.0052	0.99	1.0000	0.73	0.1278
Erythritol	1.10	0.8549	1.15	0.2990	1.05	0.9163	1.06	0.7099	1.13	0.5746
Ribitol	2.23	0.0255	0.95	0.6797	0.92	0.3219	1.10	0.5281	1.00	0.8659
Tartarate	1.59	0.1017	1.11	0.5826	1.12	0.3934	1.04	0.8618	–	
Tartronate (hydroxymalonate)	1.68	0.0021	1.60	0.0161	1.03	0.6664	1.29	0.0957	0.95	0.7552
Threonate	1.86	0.0021	1.00	0.8302	1.10	0.3593	1.14	0.6778	0.85	0.1390
Ergothioneine	1.92	0.0139	1.08	0.8302	1.31	0.0432	1.25	0.3287	1.36	0.1390
Hercynine	1.99	0.1017	–		1.44	0.0267	1.42	0.6778	–	
Stachydrine	0.92	0.2850	0.64	0.0067	0.76	0.8390	0.86	0.8800	–	
N-methylpipecolate	1.13	0.6096	–		–		–		–	
3-Hydroxypyridine sulfate	3.57	0.0076	–		–		–		–	
Beta-guanidinopropanoate	–		0.97	1.0000	0.81	0.5468	0.85	1.0000	–	
Beta-sitosterol	1.40	0.0557	1.15	0.2813	–		1.76	0.0704	–	
Campesterol	1.28	0.0449	1.11	0.0992	1.29	0.0913	1.31	0.1397	1.08	0.7552
Deoxycholate	0.43	0.7171	–		0.70	0.8032	–		–	
Taurodeoxycholate	0.48	0.2850	1.23	0.5826	0.68	0.4297	0.77	0.9347	–	
6-Beta-hydroxylithocholate	0.35	0.8818	1.66	0.2813	–		–		–	
Taurolithocholate	–		1.74	0.0991	–		1.20	0.9784	–	
Ursodeoxycholate	0.18	0.1890	0.72	0.3456	0.39	0.5814	–		–	
Tauroursodeoxycholate	0.26	0.9162	0.85	0.5826	0.34	0.4669	0.62	0.1653	–	
Taurohyocholate	–		1.27	0.7303	–		–		–	
Hyodeoxycholate	–		3.51	0.0273	–		–		–	
7-Ketodeoxycholate	0.15	0.4964	0.91	0.9547	0.24	0.3934	0.59	1.0000	–	
Taurohyodeoxycholic acid	2.21	0.0905	–		–		–		–	
3-Dehydrocholate	0.09	0.8168	–		–		–		–	
12-Dehydrocholate	0.02	0.0193	–		–		–		–	
Ursocholate	2.19	0.0850	–		–		–		–	

F–C, fold change; *q*-value by Mann–Whitney U-test, followed by Benjamini–Hochberg FDR adjustment. “–” indicates not detected. N = 9 control and N = 8 alcohol-treated dams and their fetuses.

Many metabolites within maternal plasma exchange readily across the placenta and become bioavailable to the fetus. Thirty-one MDPs were detected in placenta, including plant phenolics, indoles, and secondary bile acids (Table 4). Of these, alcohol significantly altered the abundance of 11 MDPs, and seven plant-derived phenolics and several betaines were enriched, while indolepropionate and gluconate were reduced.

The placental data suggested that MDPs might circulate within the fetus. Although fetal plasma was too scant for analysis, other fetal tissues were readily characterized (Table 4). Thirty MDPs were detected in fetal liver and/or brain. The fetal liver profile largely replicated that of maternal liver, and all but five (enterolactone sulfate and four secondary bile acids) of the 32 metabolites present in maternal liver were also detected in fetal liver. Responses to alcohol also trended similarly, with enrichments in hippurate (2.43-fold), catechol sulfate (1.83-fold), salicylate (1.46-fold), phenol sulfate (1.34-fold), and ergothioneine (1.29-fold). Although not MDPs, the phytosterols beta-sitosterol (1.76-fold) and campesterol (1.31-fold) were also elevated.

Sixteen MDPs were detected in fetal brain (Table 4). These included plant phenolics (benzoate, hippurate, p-cresol sulfate, phenol sulfate, 4-vinylcatechol sulfate), indoles (indolelactate, indolepropionate, 3-formylindole), sugar derivatives (mannonate, gluconate, erythritol, tartronate), ergothioneine, and campesterol. Alcohol altered the abundance of several MDPs, and it elevated hippurate (1.82-fold), phenol sulfate (2.72-fold), and ergothioneine (1.36-fold), and reduced p-cresol sulfate (0.81-fold) and gluconate (0.73-fold). In contrast to maternal plasma, MDPs did not explain exposure-related variance in the T-statistic (Fig. 5b,c,d), and they comprised few of the metabolites having significantly altered representation in placenta (11/116), fetal liver (2/29), and fetal brain (1/54).

## Discussion

The enteric microbiome generates a complex spectrum of biochemicals that have a substantial influence on the host^14–17^. This is the first study to document that PAE significantly alters this biochemical profile within the maternal–fetal dyad. This metabolite profile is derived from and influenced by the composition of the enteric microbiota, and the changes documented here are consistent with alcohol’s known ability to alter that composition^36,37^. Growing evidence demonstrates that microbe-derived products are mechanistic in the pathologies that underlie alcohol-related organ damage^33–35^, and our findings suggest that parallel mechanisms may operate during PAE with similar pathological consequences. Importantly, we show that these biochemicals cross the placenta and circulate within the fetus, where they could directly impact development. Indeed, many of these biochemicals having significantly altered representation, mostly plant-derived phenolics and secondary bile acids, have well-documented effects upon host physiological and cellular processes^16,18,44,45^. Our demonstration that alcohol alters their abundance within both mother and fetus introduces a novel mechanism by which PAE could alter fetal development, and thus these findings have clinical relevance.

Moreover, these biochemicals form a plasma biosignature that distinguishes the PAE pregnancies. There is substantial interest in identifying biomarkers of alcohol exposure as these enable clinicians to focus interventions on those pregnancies at greatest risk. In addition to the established markers phosphatidylethanol and ethyl-glucuronide^8,9^, recent studies have identified microRNA^46^ and cytokine-chemokine^47^ plasma signatures that may be selective for alcohol-exposed pregnancies. An MDP signature could complement those measures, as some features were enriched seven- to 13-fold in this model. Although the composition of any microbial biosignature is shaped by considerations including diet, the enteric community’s taxonomic structure, exome profile, and host species and sex^15,48,49^, our data lend proof-of-concept for the existence of such a biosignature that may complement existing markers and further enhance their specificity to detect PAE.

How might these microbial metabolites impact fetal development and alcohol-related pathologies? In this study, the dominant microbial compounds enriched by alcohol were plant-derived aromatics, mostly phenolic acids that originate from the fermentation of ingested lignin bedding and starch-bound flavonoids^18,43,50^. These same phytochemicals are abundant in edible plants, and the human enterocyte and microbiota have similar capabilities to release, convert, and absorb these compounds^18^. Humans consume an estimated two-plus grams daily of plant phytochemicals from foods and beverages, and plasma levels typically range in the nanomolar to low micromolar range^51,52^. This is the first report that these compounds circulate within the fetus. These compounds have a short half-life due to their rapid excretion^44^; that alcohol enriched both their aglycone and conjugated forms suggests that it enhanced and/or prolonged their enteric metabolism and intestinal absorption, as well as their phase II conversion. In human studies, these compounds are typically associated with improved health outcomes and reduced all-cause mortality. Mechanistically, phenolic acids improve vascular tone through stimulation of endothelial Nrf2 and nitric oxide signaling, and have anti-inflammatory actions through their inhibition of pro-oxidant enzyme-signaling cascades^18,44^; thus, their elevation in PAE may potentially mitigate some of alcohol’s damage to the mother-fetal dyad. They also act as prebiotics and directly alter the microbiome composition^52^. Highly processed Western-style diets are low in lignins and flavonoids, and their enrichment here represent a novel means by which diet may modulate FASD outcome.

The microbiota-derived secondary bile acids also defined the ALC dams, but were negative drivers within the Principal Components and Pearson Correlation analyses. Along with their parent primary bile acids, they comprised a correlated network that suggests a shared mechanistic response to PAE. Bile acids and the enteric microbiota operate in a two-way interaction that governs both bile acid metabolism and microbiota composition^45,53^. Our data suggest that alcohol altered that regulatory relationship, and this is consistent with its known effects on host-microbial bile acid pools, wherein chronic alcohol abuse elevates secondary bile acid levels^54,55^, perhaps through dysregulation of hepatic bile acid synthesis^56^. The reductions here may reflect our shorter exposure (days vs. months) and perhaps influences from the pregnancy state^57^. We could not infer which microbial populations mediated these reductions because secondary bile acid metabolism is redundant across phyla^45^. The elevated taurine conjugates in the alcohol-exposed maternal liver implicate reduced microbial deconjugation and/or hepatic amidation as additional modifying mechanisms. Secondary bile acids modulate numerous processes. They stimulate the production of antimicrobial peptides that suppress the growth of proinflammatory, gram-negative microbes^58,59^, and their reductions here suggest a means by which alcohol promotes the proinflammatory environment that worsens fetal development^47,60^. Bile acid interactions with their RXR, FXR, LXRα, and GPBAR1 receptors affect insulin sensitivity, adiposity, and lipid metabolism, conditions that independently worsen gestational outcomes^61^. Secondary bile acids were recently detected in the porcine fetus^62^, suggesting their fetal presence is not unique to rodents; however, any biological impact upon fetal development is currently unknown. Taken together, these data suggest that alcohol disturbs microbiota—bile acid interactions in a manner that could negatively impact maternal–fetal health.

Additional MDPs altered by PAE included indoles and betaine derivatives. The betaine-like compounds ergothionine and hercyine scavenge free radicals and reduce oxidative damage^63^, and their elevation in PAE may confer some protection. Indoles are generated by microbial tryptophanase and can influence brain and behavior. Oxindole, which was elevated in plasma from ALC dams, promotes anxiety-like behaviors in the open field and elevated plus-maze tests in rats^26^, behaviors also seen in PAE. Conversely, indolepropionate confers protection against neuroinflammation and TLR4 signaling through interactions with the microglial arylhydrocarbon receptor^22,28^, and sustains gut integrity through the pregnane X receptor (PXR)^64^; its sharp reduction in ALC plasma and placenta is consistent with alcohol’s pro-inflammatory actions^38,60^. Indoles also compete with amino acid and neurotransmitter efflux at the blood–brain barrier, and are functionally linked with anxiety, depression, cognitive impairment, and Parkinson’s disease^20,26,65,66^. As physiologically relevant agonists for the arylhydrocarbon receptor, they modulate not only immune function but also xenobiotic responses and insulin sensitivity^67^. We detected at least four indoles in fetal brain (indoleacetate, indolelactate, indolepropionate, 3-formylindole), and because many indole derivatives have yet to be characterized functionally, their impact upon neurodevelopment merits additional investigation.

This study has several important limitations. The first is that not all MDPs could be investigated. Many remain unannotated and likely comprise some of the unknown metabolites detected here. We also did not analyze the gut microbiota, and thus do not know if and how alcohol affects its composition in pregnancy. This study was not designed to distinguish those metabolites having a dual microbial-host origin, such as lipids, organic acids, and polyamines, nor does the methodology detect the larger MDPs that contribute to alcohol’s proinflammatory actions, such as LPS^33,38,64^. Finally, we cannot distinguish the relative contributions of enteric synthesis and cecal permeability to the elevated MDP abundance. As alcohol promotes both dysbiosis and gut permeability^33–38^, both mechanisms likely contribute; additional studies will inform this question.

In summary, alcohol alters the maternal plasma MDP profile, and by inference perhaps the microbiota composition that produced them. Several of the MDPs elevated by PAE (catechol sulfate, 4-ethylphenylsulfate, erythritol, indolepropionate, oxindole, p-cresol, salicylate) have been implicated in neuroinflammation, depression, anxiety, and autism^22,26,28,30,65,66^, outcomes also characteristic for PAE^1,2^. Other compounds may confer benefits through effects on vascular tone and anti-inflammatory actions, and thus could mitigate some of alcohol’s damage to the fetus. These MDPs circulate within the fetus, where their impact is unknown. Their enrichment, particularly in phenolic acids, constitutes a characteristic biosignature that distinguishes the PAE pregnancies, and their enrichment might also signal the presence of proinflammatory MDPs such as LPS. Together, these data suggest the novel hypothesis that the maternal microbiome may be an important mechanistic driver in the pathologies that underlie FASD.

## Methods

### Animal husbandry and alcohol-exposure

Five-week-old C57BL/6 J female mice (Jackson Laboratories, Bar Harbor, ME) were housed as pairs or trios in ventilated cages on aspen chip bedding (Northeastern Products Corp, Warrensburg NY) and cotton nesting material (Nestlets, Ancare, Bellmore NY), and a 12 h light/dark cycle (lights on 7am). Mice consumed the fixed-nutrient, purified diet AIN-93G (TD.94045, Envigo-Teklad, Madison WI^68^) throughout the study; its composition is provided in Supplementary Table S4 online. At age 8 weeks, mice were mated overnight to C57BL/6 J males. The morning of vaginal plug detection was defined as E0.5. On E8.5, pregnant females received either 3 g/kg alcohol (ALC; USP grade) or isocaloric maltodextrin (control; LoDex-10; #160175, Envigo-Teklad) once daily (9am) through E17.5 via oral gavage. Experimental group was assigned on E8.5 using a random number generator (Excel), and mice were cohoused by treatment with those sharing the same plug date. Four hours after the gavage on E17.5, mice were killed by isoflurane overdose and their tissues were flash-frozen for analysis. Blood alcohol concentrations were quantified using oxometry (Analox GM7; London, UK), according to the manufacturer’s protocol. Studies were approved by the Animal Care and Use Committee of the David H. Murdoch Research Institute, and were performed in accordance with relevant guidelines and regulations.

### Experimental blocking

We evaluated maternal plasma, maternal liver, placenta (with decidua removed), fetal liver, and fetal brain, from 9 Control and 9 ALC dams and their litters. To obtain sufficient fetal tissue for analysis, it was necessary to pool the fetuses. Specifically, for each litter, we held uterine position constant and defined Fetus 1 as occupying the position closest to the right ovary. Fetuses were numbered consecutively thereafter. Selecting fetuses 1 through 4, we combined half of fetal livers 1 through 4, and half of fetal brains 1 through 4, and submitted each pooled sample for metabolome analysis. Thus, each dam is an individual biological sample, and each fetal sample is the pool from Fetus 1, 2, 3, and 4. Each placental sample was derived from half of placental 1 and 2, because this tissue was larger. For each group (ALC, Control), we subjected nine individual dams and nine fetal pools to metabolome analysis.

### Metabolite analysis

Untargeted metabolite analysis was performed by Metabolon (Morrisville, NC), and their detailed methods are presented in Supplemental Methods S1. To summarize, samples were treated with methanol to remove protein, and then divided into five aliquots for reverse phase (RP)/UPLC–MS/MS with positive ion mode electrospray ionization (ESI, 2 samples), RP/UPLC–MS/MS with negative ion mode ESI (one sample), and HILIC/UPLC–MS/MS with negative ion mode ESI (one sample); a fifth sample was reserved for back-up. Quality controls include technical replicates of pooled experimental samples, extracted water and solvent blanks, addition of recovery standards to monitor variability and efficiency, and internal standards that assessed instrument variability and aided chromatographic alignment. Sampling order was randomized across each platform run. Compounds were identified by comparison to library entries of purified standards or recurrent unknown entities. Identification was based on the criteria of retention time/index, match to a mass to charge ratio ± 10 ppm, and chromatographic data (MS/MS spectrum). Proprietary visualization and interpretation software were used to confirm peak identities. Peaks were quantified using area-under-the-curve.

### Statistical analyses of metabolites

In the initial analysis, we tested for unequal variance between the Control and ALC groups using Shapiro-Wilks test, and tested for normality using the Levine’s test, followed by analysis for significance using the Mann–Whitney U-test. For values that were missing, we imputed the minimum value obtained for that metabolite in that tissue, and Supplemental Table S5 presents the raw LC–MS/MS dataset, as provided by Metabolon. *P*-values were adjusted for multiple testing correction using the Benjamini–Hochberg False Discovery Rate (FDR) correction^69^, and are presented as *q*-values. Analyses were performed in ArrayStudio on log transformed data^70^. For analyses that were not standard within ArrayStudio, the program R (version 3.6.1)^71^) was used. Fold-change was determined as the difference between group averages, placing ALC in the numerator, and is reported in log2 values.

For the discriminant analysis between ALC and Controls, the plasma data were scaled to a zero mean with a standard deviation of one for each metabolite, and were then run through multivariate analysis. Principal Component Analysis (PCA) was performed using FactoMineR (version 2.3)^72^ to test for separation between the treatment and control groups, and to identify outliers and question trends that supported class separation of the experimental design; findings were visualized using Factoextra R (version 1.0.7)^73^. Metabolite-metabolite correlation analysis was calculated using Spearman’s correlation on un-scaled data, and were analyzed and visualized in ggplot2 (version 3.3.0)^74^ using hierarchical clustering for comparison with Hierarchical Clustering of Principal Components (HCPC). The metabolite-metabolite correlation matrix was used to construct a network visualization to explore similarly affected metabolites. The correlation networks were constructed in Cytoscape (version 3.7.2)^75^ using the RCy3 package (version 2.6.3)^76^ and aMatReader (version 1.1.3)^77^. Only Spearman’s correlations above 0.9 were included in the network, and nodes were overlaid with descriptive statistics including q-value and log-fold change (logFC). To evaluate metabolites acting in concert, we explored the loadings plot of the sample PCA again using FactoMineR on transposed scaled data and visualized in factoextra. Hierarchical clustering analysis was used to cluster the regression factor scores of the loadings identified in the PCA using Ward’s minimum variance method and squared Euclidean distance in FactoMineR. This analysis, including the final k-means clustering, was identically applied to the correlation matrix and visualized in ggplot.

## Supplementary Information

Supplementary Information
