# Supplementary material for: An enriched biosignature of gut microbiota-dependent metabolites characterizes maternal plasma in a mouse model of fetal alcohol spectrum disorder
Source: Sci Rep. 2021 Jan 8;11:248. doi: 10.1038/s41598-020-80093-8 (PMC7794323; doi:10.1038/s41598-020-80093-8)
Supplement: Supplementary file 1 — Supplementary Information [file 41598_2020_80093_MOESM1_ESM.pdf]

Supplementary Materials for:

"An Enriched Biosignature of Gut Microbiota-Dependent Metabolites Characterizes Maternal Plasma in a Mouse Model of Fetal Alcohol Spectrum Disorder"

Manjot S. Virdee, Nipun Saini, Colin D. Kay, Andrew P. Neilson, Sze Ting Cecilia Kwan, Kaylee K. Helfrich, Sandra M. Mooney, and Susan M. Smith

Supplementary Table S1.
Maternal-Fetal Growth and Survival Characteristics

	Controls	Alcohol-Exposed	*p*-Value
Maternal Weight at E0.5, g	18.37 ± 1.18	18.16 ± 1.09	0.71
Maternal Weight at E8.5, g	21.20 ± 1.68	21.10 ± 1.36	0.88
Maternal Weight Gain (E0.5-E17.5), g	13.96 ± 2.23	13.28 ± 2.25	0.52
Maternal Weight Gain (E8.5-E17.5), g	11.08 ± 1.50	10.35 ± 1.47	0.28
Litter Size at E17.5	7.00 ± 1.22	7.11 ± 1.36	0.85
Fetal Weight at E17.5, g	0.92 ± 0.11	0.88 ± 0.12	0.31
Resorptions at E17.5	1.00 ± 1.00	1.00 ± 1.22	0.81
Percent Survival at E17.5 *	88.8 ± 3.58	84.7 ± 4.57	0.69

All values are mean ± SD. with N=9 dams per treatment group. Statistical comparisons using ANOVA (for normal data with equal variance) or Kruskal-Wallis test (for non-normal data and/or data with unequal variance).

*Percent Survival calculated as [Number of live fetuses / (number of live fetuses + number of resorptions)] × 100.

Supplementary Table S2.
Microbial-Derived Products that Drive Dimensions 1 and 2 in the Hierarchical Clustering.

Metabolite	F-C	Q-Value	PCA 1	PCA 2
**Cluster 1**				
2,6-dihydroxybenzoic acid	2.54	0.0021	0.7752688	-0.4351092
2-hydroxyhippurate (salicylurate)	2.91	0.0277	0.7584338	-0.0953286
3-(3-hydroxyphenyl)propionate sulfate	2.61	0.0462	0.5634596	-0.1519389
3-(4-hydroxyphenyl)propionate	3.45	0.0244	0.5751308	-0.4839515
3-ethylphenylsulfate	2.05	0.0076	0.6555667	-0.5171646
3-formylindole	1.50	0.0021	0.8332612	-0.2511513
3-hydroxypyridine sulfate	3.57	0.0076	0.7443017	-0.3851936
3-indoleglyoxylic acid	1.48	0.0139	0.7904930	-0.0868398
3-phenylpropionate (hydrocinnamate)	3.69	0.0076	0.5436052	-0.3792856
4-acetylphenyl sulfate	7.69	0.0066	0.7675759	-0.2719357
4-allylphenol sulfate	2.53	0.0680	0.7526993	0.1000044
4-ethylphenol glucuronide	3.28	0.0076	0.8375959	-0.3614704
4-ethylphenyl sulfate	2.11	0.0255	0.8198300	-0.2521090
4-hydroxycinnamate	7.23	0.0021	0.8539094	-0.3314298
4-hydroxycinnamate sulfate	7.57	0.0076	0.8518206	-0.2591238
4-methylcatechol sulfate	3.11	0.0030	0.7300599	-0.2779232
4-vinylcatechol sulfate	3.91	0.0021	0.7556210	-0.4518426
4-vinylphenol sulfate	1.91	0.0100	0.8585357	-0.1534378
beta-sitosterol	1.40	0.0557	0.7709181	0.0538341
caffeic acid sulfate	3.21	0.0021	0.8487926	-0.3407625
campesterol	1.28	0.0449	0.8448519	0.1240419
catechol sulfate	7.41	0.0021	0.8673520	-0.2944675
cinnamate	5.28	0.0076	0.7580338	-0.2742997
enterolactone (isocitric lactone)	1.64	0.0139	0.7673101	-0.2049301
enterolactone sulfate	5.32	0.0183	0.7221703	0.0233456
ergothioneine	1.92	0.0139	0.6723721	0.1089141
ferulic acid 4-sulfate	4.50	0.0076	0.7897056	-0.3431169
gentisate	3.99	0.0021	0.9273447	-0.0504719
gluconate	1.39	0.2131	0.5059782	-0.4340679
hippurate	3.37	0.0021	0.8759632	-0.2947280
histidine betaine (hercynine)	1.99	0.1017	0.5616067	0.0500921
indoleacetate	1.46	0.0021	0.6343635	-0.4379542
methyl-4-hydroxybenzoate sulfate	3.13	0.0097	0.6557600	-0.2658955
phenol sulfate	3.20	0.0139	0.7212892	-0.1465567
salicin	12.98	0.0097	0.7885188	-0.2487080
salicylate	3.11	0.0076	0.8981055	0.1034211
tartarate	1.59	0.1017	0.4954565	-0.5476356
tartronate (hydroxymalonate)	1.68	0.0021	0.8284888	0.1832853
taurohyodeoxycholic acid	2.21	0.0905	0.2249110	-0.6240830
threonate	1.86	0.0021	0.8701176	0.1092360
ursocholate	2.19	0.0850	0.6531903	-0.0790253
**Cluster 2**				
1H-indole-7-acetic acid	1.32	0.7981	0.2464132	0.5716379

4-methylbenzenesulfonate	1.05	0.8168	0.3340250	0.0036705
methyl indole-3-acetate	0.77	0.7682	0.0765753	0.0609480
**Cluster 3**				
2-aminophenol sulfate	1.89	0.0462	0.4772276	-0.3670068
2-oxindole-3-acetate	1.32	0.3170	0.4934865	-0.2048566
4-hydroxyhippurate	2.68	0.0100	0.7770098	-0.2280412
cinnamoylglycine	4.86	0.0126	0.7325696	-0.1480215
dihydrocaffeate sulfate	1.44	0.1859	0.5188975	0.0976087
erythritol	1.10	0.8549	0.3246582	0.0443041
indolelactate	1.76	0.0021	0.6259044	-0.2141652
indolin-2-one	1.47	0.0680	0.3984721	-0.0303745
mannonate	1.21	0.8549	0.5573894	0.3145222
ribotol	2.23	0.0255	0.6099912	-0.2535124
thymol sulfate	1.96	0.4964	0.3677674	0.0843172
**Cluster 4**				
6-hydroxyindole sulfate	0.99	1.0000	-0.0924610	0.3518308
N-methylpipecolate	1.13	0.6096	0.4520928	0.6020761
p-cresol glucuronide	0.80	0.3169	-0.3530030	0.3028724
p-cresol sulfate	1.03	1.0000	0.1224484	0.3639081
stachydrine	0.92	0.2850	0.2092769	0.4994248
**Cluster 5**				
12-dehydrocholate	0.02	0.0193	-0.7987420	-0.2208106
3-dehydrocholate	0.09	0.8168	-0.6010296	-0.5753394
6-beta-hydroxylithocholate	0.35	0.8818	-0.3620295	-0.4266377
7-ketodeoxycholate	0.15	0.4964	-0.6458032	-0.4714987
benzoate	1.07	0.4631	-0.0176586	-0.4394711
deoxycholate	0.43	0.7171	-0.5468652	-0.4441932
glycocholate	1.47	0.3577	0.0162101	-0.5989848
indolepropionate	0.41	0.0139	-0.7083837	0.1287851
taurodeoxycholate	0.48	0.2850	-0.1696046	-0.5227686
tauroursodeoxycholate	0.26	0.9162	-0.5758796	-0.5328175
ursodeoxycholate	0.18	0.1890	-0.7154062	-0.2721035

* N=9 control, N=8 alcohol. F-C, fold-change; q-values from Mann-Whitney U-test followed by Benjamini-Hochberg correction.

Supplementary Table S3.
Microbial-Derived Products by Cluster from the Hierarchical Clustering on Spearman's Correlation.

Cluster	Metabolite	F-C	Q-Value	PCA
1	1H-indole-7-acetic acid	1.32	0.7981	0.2464132
	4-methylbenzenesulfonate	1.05	0.8168	0.3340250
	6-beta-hydroxylithocholate	0.35	0.8818	-0.3620295
	6-hydroxyindole sulfate	0.99	1.0000	-0.0924610
	benzoate	1.07	0.4631	-0.0176586
	glycocholate	1.47	0.3577	0.0162101
	mannonate	1.21	0.8549	0.5573894
	N-methylpipecolate	1.13	0.6096	0.4520928
	stachydrine	0.92	0.2850	0.2092769
	taurodeoxycholate	0.48	0.2850	-0.1696046
	taurohyodeoxycholic acid	2.21	0.0905	0.2249110
	tauroursodeoxycholate	0.26	0.9162	-0.5758796
	thymol sulfate	1.96	0.4964	0.3677674
2	methyl indole-3-acetate	0.77	0.7682	0.0765753
	p-cresol glucuronide	0.80	0.3169	-0.3530030
	p-cresol sulfate	1.03	1.0000	0.1224484
3	3-dehydrocholate	0.09	0.8168	-0.6010296
	7-ketodeoxycholate	0.15	0.4964	-0.6458032
	deoxycholate	0.43	0.7171	-0.5468652
	hercynine	1.99	0.1017	0.5616067
	ursodeoxycholate	0.18	0.1890	-0.7154062
4	2-aminophenol sulfate	1.89	0.0462	0.4772276
	2-oxindole-3-acetate	1.32	0.3170	0.4934865
	dihydrocaffeate sulfate	1.44	0.1859	0.5188975
	erythritol	1.10	0.8549	0.3246582
	indolin-2-one	1.47	0.0680	0.3984721
5	12-dehydrocholate	0.02	0.0193	-0.7987420
	2,6-dihydroxybenzoic acid	2.54	0.0021	0.7752689
	2-hydroxyhippurate (salicylurate)	2.91	0.0277	0.7584338
	3-(3-hydroxyphenyl)propionate sulfate	2.61	0.0462	0.5634596
	3-(4-hydroxyphenyl)propionate	3.45	0.0244	0.5751308
	3-ethylphenylsulfate	2.05	0.0076	0.6555667
	3-formylindole	1.50	0.0021	0.8332612
	3-hydroxypyridine sulfate	3.57	0.0076	0.7443017
	3-indoleglyoxylic acid	1.48	0.0139	0.7904930
	3-phenylpropionate (hydrocinnamate)	3.69	0.0076	0.5436053
	4-acetylphenyl sulfate	7.69	0.0066	0.7675759
	4-allylphenol sulfate	2.53	0.0680	0.7526993
	4-ethylphenol glucuronide	3.28	0.0076	0.8375959
	4-ethylphenyl sulfate	2.11	0.0255	0.8198300
	4-hydroxycinnamate	7.23	0.0021	0.8539094
	4-hydroxycinnamate sulfate	7.57	0.0076	0.8518207
	4-hydroxyhippurate	2.68	0.0100	0.7770098
	4-methylcatechol sulfate	3.11	0.0030	0.7300599
	4-vinylcatechol sulfate	3.91	0.0021	0.7556210

4-vinylphenol sulfate	1.91	0.0100	0.8585357
beta-sitosterol	1.40	0.0557	0.7709181
caffeic acid sulfate	3.21	0.0021	0.8487926
campesterol	1.28	0.0449	0.8448519
catechol sulfate	7.41	0.0021	0.8673521
cinnamate	5.28	0.0076	0.7580338
cinnamoylglycine	4.86	0.0126	0.7325696
enterolactone	1.64	0.0139	0.7673101
enterolactone sulfate	5.32	0.0183	0.7221703
ergothioneine	1.92	0.0139	0.6723721
ferulic acid 4-sulfate	4.50	0.0076	0.7897056
gentisate	3.99	0.0021	0.9273448
gluconate	1.39	0.2131	0.5059782
hippurate	3.37	0.0021	0.8759632
indoleacetate	1.46	0.0021	0.6343635
indolelactate	1.76	0.9921	0.6259044
indolepropionate	0.41	0.0139	-0.7083837
methyl-4-hydroxybenzoate sulfate	3.13	0.0097	0.6557600
phenol sulfate	3.20	0.0139	0.7212892
ribitol	2.23	0.0255	0.6099912
salicin	12.98	0.0097	0.7885188
salicylate	3.11	0.0076	0.8981055
tartarate	1.59	0.1017	0.4954565
tartronate (hydroxymalonate)	1.68	0.1017	0.8284888
threonate	1.86	0.0021	0.8701177
ursocholate	2.19	0.0850	0.6531904

* N=9 control, N=8 alcohol. F-C, fold-change; q-values from Mann-Whitney U-test followed by Benjamini-Hochberg correction.

Supplementary Table S4.
Composition of Rodent Diet AIN-93G

Ingredient	g/kg diet
Casein	200.0
L-Cystine	3.0
Corn Starch	397.486
Maltodextrin	132.0
Sucrose	100.0
Soybean Oil	70.0
Cellulose	50.0
Mineral Mix*	35.0
Vitamin Mix**	10.0
Choline Bitartrate	2.5
TBHQ	0.014

* AIN-93G-MX (Envigo #94046), [68]
** AIN-93-VX (Envigo #94047), [68]

Supplemental Table S5.
Raw Area-Under-the-Curve Values from LC-MS/MS for Plasma MDPs

	MD	MD	MD	MD	MD	MD	MD	MD	MD	ET	ET	ET	ET	ET	ET	ET	ET	ET
12-dehydrocholate	31780	511929	152421	67391	141619	332768	118934	3945842	8589015							94362		
1H-indole-7-acetic acid	66519	79718	79976	64392	78709	108135	244451		61219	226024	58988	196587	53374	29116	211677	152838	120796	112555
2,6-dihydroxybenzoic acid	5956041	4919603	8284409	6858124	5652205	1429510	2598444	4607429	5020384	1E+07	1.7E+07	1.2E+07	1E+07	1.1E+07	6607169	1.2E+07	1.6E+07	1.6E+07
2-aminophenol sulfate	25659	28930			29343				44788		77176	41466	74344	79682		32064	30232	70453
2-hydroxyhippurate (salicylurate)	165354	137794	127409	117899		101037				117542	636151	532194	287473	134019	120495	205651	303188	508089
2-oxindole-3-acetate	34982	52478								47832	93470		50028	34905		45350		32624
3-(3-hydroxyphenyl)propionate sulfate	121465									77504	237987	449286		87223	280153	398981	173060	220591
3-(4-hydroxyphenyl)propionate	177872	76174	58087			51123		117309	100145	110684	506500	371208	161719	150464	207376	468978	406199	141321
3-dehydrocholate		219876						2029490	6081718		45843			325313	254675			79846
3-ethylphenylsulfate	91110	178022	144522	208010	153622	24654	46732	128424	108916	309367	286050	293547	194778	193237	150530	360228	244956	135385
3-formylindole	6773664	6991912	6519241	7386960	8128371	4621887	5615429	5697492	6023743	9682226	9418315	9145335	9731994	8819604	7402878	9071471	1.1E+07	1.1E+07
3-hydroxypyridine sulfate	59170		23643	40012						50239	210554	76826	63303	138982	34853	126024	89959	83515
3-indoleglyoxylic acid	4897232	5539288	5749382	6112914	5373546	3094595	3966992	4687015	3076247	6694445	6308653	6534848	6784509	5361843	5265080	8085267	8365877	9054063
3-phenylpropionate (hydrocinnamate)	825245	555791	425597	342644	641807	303865	347381	679423	918773	1679587	853055	2514447	812066	1550774	2421855	2242551	4161028	3092689
4-acetylphenyl sulfate			45066	62064						95173	383740	320819	135165	245146	35334	155728	822132	264731
4-allylphenol sulfate	333244	18994	545301	652285	105523	35181	283211			560484	620863	498347	564072	500976	424811	577733	524188	682634
4-ethylphenol glucuronide	62691	66982	29306	82796	40184			31066	44781	103970	207608	109699	177704	170946	35685	158171	146932	155223
4-ethylphenyl sulfate	1414286	883991	1109212	1103682	800922	551525	397120	529522	410239	1554094	1886967	2157004	1251659	1114579	828542	2589555	1899824	1246799
4-hydroxycinnamate	372778	556590	740908	891314	394109	88411	193689	201820	274740	1168316	7073269	2082850	1539825	1947143	774665	1806426	6541803	1940237
4-hydroxycinnamate sulfate	143493	157228	118831	157503	43621		46508		63741	270355	1434091	445661	308109	330375	168474	426094	1667070	624325
4-hydroxyhippurate	210963	199280	234144	260279	92756	143377	131279	56024	142283	164472	1033198	387070	328239	465450	157751	388392	387343	376426
4-methylbenzenesulfonate	107247	190472	122204	122069	141665	111312	112550	99486	115334	104085	140762	135368	110262	117801	216121	141093	179454	132159
4-methylcatechol sulfate	1320161	1195566	1413895	1468969	1051333	606729	737699	734371	2223912	2455975	4071574	4488686	2425994	1976065	1289077	2618264	3258915	8792899
4-vinylphenol sulfate	4765664	6556914	6837310	6013398	6251994	2435606	3551484	3263967	3241354	8454054	1.1E+07	1E+07	7417701	5068683	7042345	1.2E+07	1.2E+07	7953135
6-beta-hydroxylithocholate	52442	270343	27483	30854	43489	52548	243939	1833942	860401	137775	188927	115159	92363	206388	429711	111640	85002	221554
6-hydroxyindole sulfate	425586	265257	324828	189765	290236	328251	338221	236447	434381	350707	193418	302510	468834	151768	181976	273793	323387	453368
7-ketodeoxycholate	741904	3267286	1171035	1373590	534002	1006299	2545004	1.9E+07	5.7E+07	1086377	1491799	789715	508903	5447327	4792829	656294	556013	1458989
benzoate	1103788	958546	1076344	1119065	915383	790408	787373	773648	3410531	1043758	1057396	2997122	921650	1127451	2259897	1054840	1235650	1100601
beta-sitosterol	519630	916622	723176	1058129	966360	797850	576867	529332	604112	983751	1184387	896579	1006009	746108	1198193	1004775	1317331	1280838
caffeic acid sulfate	7895971	1.1E+07	1.3E+07	1.3E+07	6861479	1493983	3437946	5020427	4115817	1.7E+07	4.7E+07	2.4E+07	1.7E+07	1.6E+07	8182893	2.1E+07	2.8E+07	2.1E+07
campesterol	1765413	2425338	2523457	2650680	2268993	1996994	1720272	1217846	1584833	2819843	2464545	2311536	2949886	2294494	2747627	2570634	2713529	2784382
catechol sulfate	739768	588707	1437283	1852112	462859	235152	350126	283681	491694	2685736	9745918	5150721	3572512	4597983	1988479	3001630	7409037	6577375
cinnamate	135736	59687	50826	70128	55289	49915	32790		89779	248185	187036	408253	186974	250716	140830	334618	554604	536088
cinnamoylglycine	283450	487156	185328	158653	218476	196166	157400		149417	347322	714568	738057	1252416	1421789	271932	466727	796887	2841726
deoxycholate	5647098	7721930	7966184	2941720	3442629	2270060	1E+07	5.5E+07	1.7E+07	8418536	5524379	3444857	3970882	1.4E+07	1.4E+07	3336990	2556730	5454383
dihydrocaffeate sulfate (2)	198254	170392	94011	86175	124031	148088	101364	27724	154592	66866	236196	155643	198926	151075	47595	192915	160883	262384
ergothioneine	1324094	1032673	1234194	1317299	1875191	616492	2057902	882660	900387	2540301	1883784	3620752	1383838	1420229	1043662	2168522	2885499	3503929
erythritol	354689	445751	403195	421249	298338	386441	368910	178783	477703	320785	476339	394332	620889	460396	246786	293987	308276	420166
ferulic acid 4-sulfate	158146	167340	63599			41872			64588	183098	723526	269952	272004	247754	170122	296677	440687	219793
gentisate	449893	431559	436763	549903	206914	174627	225041	107704	97485	809016	1910752	1558067	859090	566499	597651	1044277	1406863	1406066
gluconate	1616638	1547820	1392815	3093537	1554023	1117789	1179554	1283625	2202543	2009985	2589325	1843977	2146263	1964180	1225575	3074369	3857312	1328149
glycocholate		164976		34760		25936	112216	750686	747250	435818	603875	85981	60298	742946	752850	177282	159085	401035
hippurate	3395526	5744682	6596183	5208238	3157197	1779322	1418156	1507298	3145428	7919662	1.6E+07	1.6E+07	1E+07	8174459	4678695	9494354	1.6E+07	1.3E+07
histidine betaine (hercynine)*					340969					226331	233214	312200	77255			146931	227104	399046
indoleacetate	1279300	1162571	1124185	1200909	887598	1122987	895200	1054389	1097529	1731084	1322141	1436428	1815294	1699994	1145071	1862294	1631427	1472746
indolelactate	2.9E+07	2E+07	3.3E+07	3E+07	2.5E+07	2.6E+07	3.3E+07	2.2E+07	3.7E+07	4.9E+07	4.8E+07	3.7E+07	7E+07	5.3E+07	3.1E+07	4.6E+07	4E+07	6.2E+07
indolepropionate	1.1E+07	1.3E+07	8182075	1.2E+07	2.5E+07	3.1E+07	1.2E+07	1.4E+07	3.1E+07	5415085	6923919	9160993	2291558	6406801	5142365	6410990	9120683	1.2E+07
indolin-2-one	380677	445174	276628	216685	283327	312091	596526	261318	229682	582728	521338	472524	381044	778028	1112319	370618	421890	465461
isocitric lactone	1275800	1357719	887981	1316662	870795	622906	648850	380538	978963	1662375	1397182	1746880	2386666	1338327	409599	1476385	1368965	904331

mannonate*	7918888	7550331	4888171	8034779	6103057	5094990	7852097	2608428	4982162	6355324	7448460	7847582	1.3E+07	7221596	2960030	5250180	4827649	7737912
methyl indole-3-acetate	767521	613317	731266	974779	3367185	299362	601302	542562	1027442	1386327	917539	1222700	461125	278889	1079609	592418	576498	703570
methyl-4-hydroxybenzoate sulfate	20506									73411	119757	38998		32612		100450	79191	49000
N-methylpipecolate	547122	445215	472305	570748	429966	627064	861723	172887	380129	468037	549495	528741	822173	526685	358908	452674	554550	660222
p-cresol glucuronide*	6371945	6166874	8639573	4946464	5311396	6041844	1.1E+07	4482697	1E+07	3837436	4917901	3558334	7321320	6179428	1066906	3883283	2748669	1.3E+07
p-cresol sulfate	1.4E+08	1.3E+08	1.8E+08	1.1E+08	1.3E+08	1.1E+08	1.9E+08	7.8E+07	1.5E+08	1.2E+08	1.3E+08	1.3E+08	1.7E+08	1.4E+08	5.3E+07	1.3E+08	9.8E+07	2.3E+08
phenol sulfate	1.3E+07	1962269	7675539	3424654	7786109	1986426	4189223	3614404	4458544	1.6E+07	2.2E+07	1.2E+07	2.2E+07	6877995	5823281	1.5E+07	1.2E+07	3.2E+07
ribitol	466906	517181	540469	544967	497952	404370	427362	428631	573894	525863	2497297	695168	1415869	996199	356783	534151	556707	1669226
salicin		25468	83702	142524						111189	1477019	422842	124486	303483	85153	230268	1381810	615109
salicylate	4203778	2279161	2516090	4622484	1762834	1787908	2145020	556251	558721	4562691	1.1E+07	8717915	4657727	3406306	5939567	6935151	8568354	8467040
stachydrine	1029393	2250072	1530627	1458497	1054093	2507366	1.2E+07	1133312	958952	2141080	4068865	2282114	2946094	1357213	3126798	3438069	1596936	2024468
tartarate	1.3E+07	3.1E+07	3.1E+07	2.3E+07	2.3E+07	2393263	5093012	2E+07	1.8E+07	3.7E+07	2.9E+07	3.8E+07	2.4E+07	2.4E+07	1E+07	3.6E+07	4.1E+07	1.1E+07
tartronate (hydroxymalonate)	1577387	1515691	1635180	1656402	1397505	1320000	2076409	817087	962973	2451397	2005796	2449840	2320434	2139720	1682196	2433366	3131725	2626018
taurodeoxycholate	613932	1226839	1300047	1011012	1010064	593283	4166009	5.1E+07	4730597	5880272	4612142	2079718	3415961	8544434	1.3E+07	2650306	1739603	2821404
taurohyodeoxycholic acid		221564		85558			341476	5032702	1902788	3715707	3169665	3308343	1360025	2068184	1.7E+07	1773751	1479680	443676
tauroursodeoxycholate	1932075	2770167	2252541	2818460	2714308	3854375	5199001	1E+08	2.6E+07	4236255	4396580	2291170	2426650	1.3E+07	1.9E+07	3474642	3439031	4268309
threonate	4.4E+08	4.8E+08	4E+08	4.7E+08	3.9E+08	3.3E+08	5.2E+08	1.9E+08	2.6E+08	7E+08	6E+08	7.2E+08	8.1E+08	7.2E+08	4E+08	6.2E+08	8.6E+08	8E+08
thymol sulfate		53029				48567			30334	35304	110021	24428			59685	47239	30303	189907
ursocholate	100831	747211				90145				681763	368247	500972	457747		3190594	321372	302764	151701
ursodeoxycholate	2285518	4009393	1693780	2007796	899439	1592158	4852579	1.9E+07	3.6E+07	1233639	1820017	941602		2989353	6370950	1047473	285887	3388112
enterolactone sulfate	155734	197523	156659	26784	34370	40070	53390	25841	43661	781812	325131	301943	194166	42749	148998	260013	882398	705020
4-vinylcatechol sulfate	219774	629837	434712	392131	332229	56811	89083	214396	237347	553062	1111716	974462	483552	568204	315816	710948	985011	579834

Supplementary Figure S1. Analysis of plasma metabolites including mouse ALC-6. **(a)** Principal component analysis places ALC-6 as an outlier that clusters within a subset of control dams. Microbial metabolite loadings are plotted against components one and two, as per Figure 1b. **(b)** K-means clustering places ALC-6 as an outlier that clusters within a subset of control dams. **(c)** PCA mapping housing assignment on each mouse identity, showing that cage assignment did not significantly affect analyte outcome. N=9 control (CON) and N=9 alcohol-exposed (ALC) dams for (S1a,b), and N=8 ALC dams for (S1c). Analyzed using FactoMineR (v.2.3) and visualized using FactoextraR (v.1.0.7).

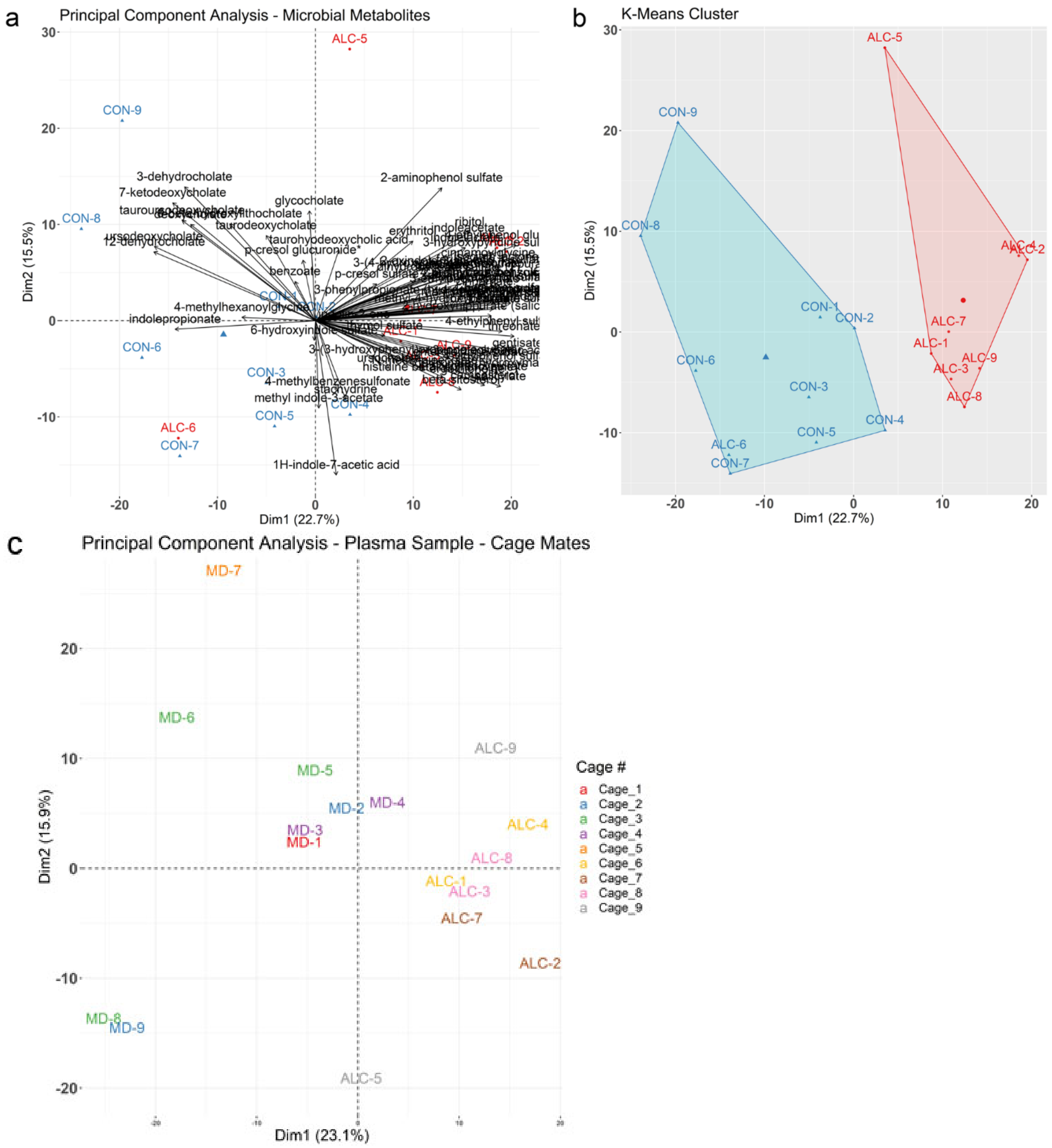

Supplementary Figure S2. Hierarchical clustering of the sample PCA loadings for metabolites in maternal plasma, plotted against the principal component score for Dimension 2 (presented in Supplemental Table S2). Microbial metabolites were distributed equally across the clusters, indicating they did not contribute to PCA dimension 2. Microbial metabolites are shown in red, endogenous compounds in blue, and unknown compounds in green. Visualized using ggplot (v.3.3.0).

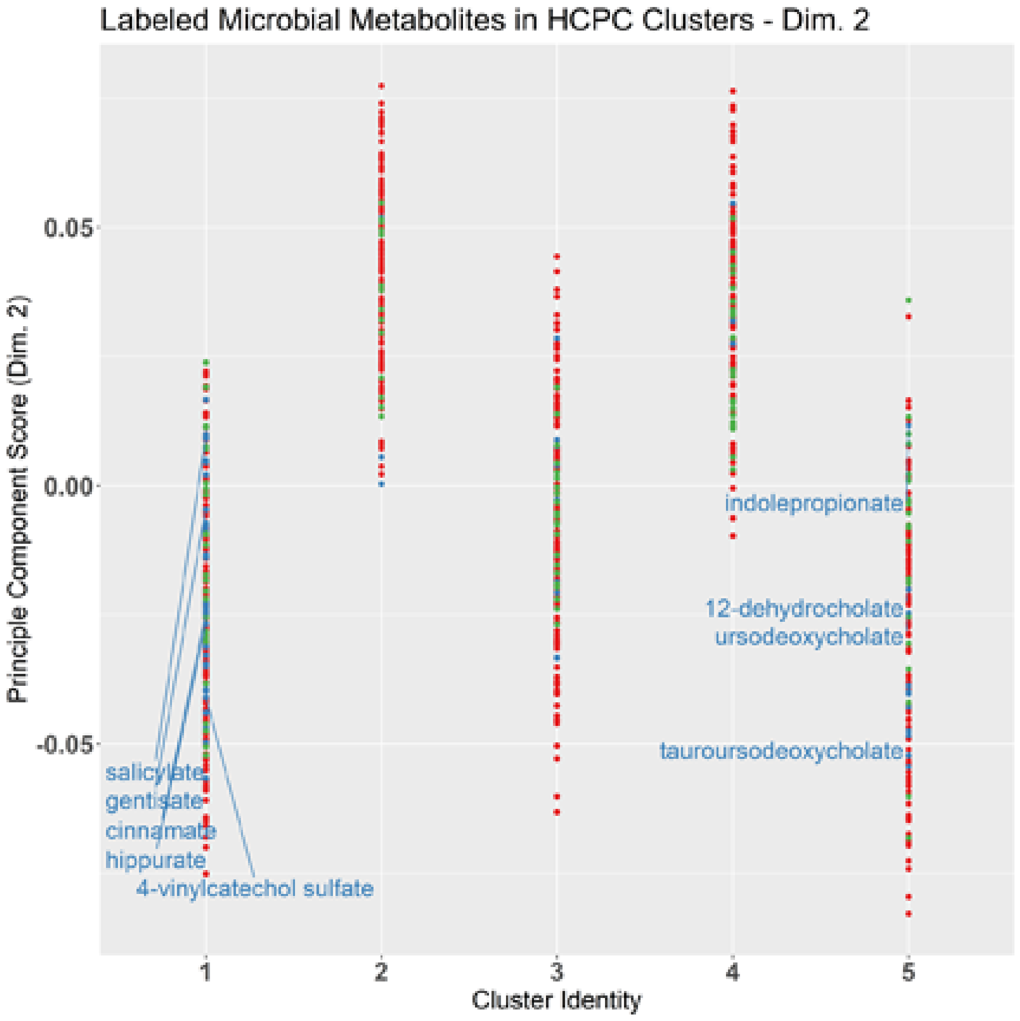

Supplemental Methods

Metabolon Platform Workflow
Citation for the untargeted metabolite analysis described below is Ramamoorthy, S. *mView Report on UNCH-06-18VW+*. Metabolon, Inc. Personal communication (October 31, 2018).

Sample Accessioning: Following receipt, samples were inventoried and immediately stored at -80°C. Each sample received was accessioned into the Metabolon LIMS system and was assigned by the LIMS a unique identifier that was associated with the original source identifier only. This identifier was used to track all sample handling, tasks, results, etc. The samples (and all derived aliquots) were tracked by the LIMS system. All portions of any sample were automatically assigned their own unique identifiers by the LIMS when a new task was created; the relationship of these samples was also tracked. All samples were maintained at -80°C until processed.

Sample Preparation: Samples were prepared using the automated Microlab STAR system from Hamilton Company. Several recovery standards were added prior to the first step in the extraction process for QC purposes. To remove protein, dissociate small molecules bound to protein or trapped in the precipitated protein matrix, and to recover chemically diverse metabolites, proteins were precipitated with methanol under vigorous shaking for 2 min (Glen Mills Geno/Grinder 2000) followed by centrifugation. The resulting extract was divided into five fractions: two for analysis by two separate reverse phase (RP)/UPLC-MS/MS methods with positive ion mode electrospray ionization (ESI), one for analysis by RP/UPLC-MS/MS with negative ion mode ESI, one for analysis by HILIC/UPLC-MS/MS with negative ion mode ESI, and one sample was reserved for backup. Samples were placed briefly on a TurboVap (Zymark) to remove the organic solvent. The sample extracts were stored overnight under nitrogen before preparation for analysis.

QA/QC: Several types of controls were analyzed in concert with the experimental samples: a pooled matrix sample generated by taking a small volume of each experimental sample (or alternatively, use of a pool of well-characterized human plasma) served as a technical replicate throughout the data set; extracted water samples served as process blanks; and a cocktail of QC standards that were carefully chosen not to interfere with the measurement of endogenous compounds were spiked into every analyzed sample, allowed instrument performance monitoring and aided chromatographic alignment. Instrument variability was determined by calculating the median relative standard deviation (RSD) for the standards that were added to each sample prior to injection into the mass spectrometers. Overall process variability was determined by calculating the median RSD for all endogenous metabolites (i.e., non-instrument standards) present in 100% of the pooled matrix samples. Experimental samples were randomized across the platform run with QC samples spaced evenly among the injections.

Ultrahigh Performance Liquid Chromatography-Tandem Mass Spectroscopy (UPLC-MS/MS): All methods utilized a Waters ACQUITY ultra-performance liquid chromatography (UPLC) and a Thermo Scientific Q-Exactive high resolution/accurate mass spectrometer interfaced with a heated electrospray ionization (HESI-II) source and Orbitrap mass analyzer operated at 35,000 mass resolution. The sample extract was dried then reconstituted in solvents

compatible to each of the four methods. Each reconstitution solvent contained a series of standards at fixed concentrations to ensure injection and chromatographic consistency. One aliquot was analyzed using acidic positive ion conditions, chromatographically optimized for more hydrophilic compounds. In this method, the extract was gradient eluted from a C18 column (Waters UPLC BEH C18-2.1x100 mm, 1.7 µm) using water and methanol, containing 0.05% perfluoropentanoic acid (PFPA) and 0.1% formic acid (FA). Another aliquot was also analyzed using acidic positive ion conditions, however it was chromatographically optimized for more hydrophobic compounds. In this method, the extract was gradient eluted from the same afore mentioned C18 column using methanol, acetonitrile, water, 0.05% PFPA and 0.01% FA and was operated at an overall higher organic content. Another aliquot was analyzed using basic negative ion optimized conditions using a separate dedicated C18 column. The basic extracts were gradient eluted from the column using methanol and water, however with 6.5mM Ammonium Bicarbonate at pH 8. The fourth aliquot was analyzed via negative ionization following elution from a HILIC column (Waters UPLC BEH Amide 2.1x150 mm, 1.7 µm) using a gradient consisting of water and acetonitrile with 10mM Ammonium Formate, pH 10.8. The MS analysis alternated between MS and data-dependent MSn scans using dynamic exclusion. The scan range varied slighted between methods but covered 70-1000 m/z. Raw data files are archived and extracted as described below.

Bioinformatics: The informatics system consisted of four major components, the Laboratory Information Management System (LIMS), the data extraction and peak-identification software, data processing tools for QC and compound identification, and a collection of information interpretation and visualization tools for use by data analysts. The hardware and software foundations for these informatics components were the LAN backbone, and a database server running Oracle 10.2.0.1 Enterprise Edition.

LIMS: The purpose of the Metabolon LIMS system was to enable fully auditable laboratory automation through a secure, easy to use, and highly specialized system. The scope of the Metabolon LIMS system encompasses sample accessioning, sample preparation and instrumental analysis and reporting and advanced data analysis. All of the subsequent software systems are grounded in the LIMS data structures. It has been modified to leverage and interface with the in-house information extraction and data visualization systems, as well as third party instrumentation and data analysis software.

Data Extraction and Compound Identification: Raw data was extracted, peak-identified and QC processed using Metabolon's hardware and software. These systems are built on a web-service platform utilizing Microsoft's .NET technologies, which run on high-performance application servers and fiber-channel storage arrays in clusters to provide active failover and load-balancing. Compounds were identified by comparison to library entries of purified standards or recurrent unknown entities. Metabolon maintains a library based on authenticated standards that contains the retention time/index (RI), mass to charge ratio (m/z), and chromatographic data (including MS/MS spectral data) on all molecules present in the library. Furthermore, biochemical identifications are based on three criteria: retention index within a narrow RI window of the proposed identification, accurate mass match to the library +/- 10 ppm, and the MS/MS forward and reverse scores between the experimental data and authentic standards. The MS/MS scores are based on a comparison of the ions present in the experimental

spectrum to the ions present in the library spectrum. While there may be similarities between these molecules based on one of these factors, the use of all three data points can be utilized to distinguish and differentiate biochemicals. More than 3300 commercially available purified standard compounds have been acquired and registered into LIMS for analysis on all platforms for determination of their analytical characteristics. Additional mass spectral entries have been created for structurally unnamed biochemicals, which have been identified by virtue of their recurrent nature (both chromatographic and mass spectral). These compounds have the potential to be identified by future acquisition of a matching purified standard or by classical structural analysis.

Curation: A variety of curation procedures were carried out to ensure that a high quality data set was made available for statistical analysis and data interpretation. The QC and curation processes were designed to ensure accurate and consistent identification of true chemical entities, and to remove those representing system artifacts, mis-assignments, and background noise. Metabolon data analysts use proprietary visualization and interpretation software to confirm the consistency of peak identification among the various samples. Library matches for each compound were checked for each sample and corrected if necessary.

Metabolite Quantification and Data Normalization: Peaks were quantified using area-under-the-curve. For studies spanning multiple days, a data normalization step was performed to correct variation resulting from instrument inter-day tuning differences. Essentially, each compound was corrected in run-day blocks by registering the medians to equal one (1.00) and normalizing each data point proportionately (termed the “block correction”). For studies that did not require more than one day of analysis, no normalization is necessary, other than for purposes of data visualization. In certain instances, biochemical data may have been normalized to an additional factor (e.g., cell counts, total protein as determined by Bradford assay, osmolality, etc.) to account for differences in metabolite levels due to differences in the amount of material present in each sample.
